# Reducing the Environmental Impact of Growing-Finishing Pig Production Through Daily Feed Adjustment: A Comparative Life Cycle Assessment

**DOI:** 10.3390/ani16101562

**Published:** 2026-05-21

**Authors:** Yann Malini, Rayna S. V. Amaral, Blandina G. V. Silva, Leila C. S. Moura, Diana A. Oliveira, Luciano Hauschild, Ines Andretta, Eduarda B. Xavier, Luis C. V. Itavo, Luan S. Santos

**Affiliations:** 1 Graduate Program in Animal Science, Universidade Federal do Rio de Janeiro, Km 07, BR-465, Seropédica 23890-000, RJ, Brazil; yannmalini@yahoo.com (Y.M.); raynamaral@ufrrj.br (R.S.V.A.); blandinagvs@gmail.com (B.G.V.S.); mouraleila@ufrrj.br (L.C.S.M.); dianaassis@ufrrj.br (D.A.O.); 2 Graduate Program in Animal Biosciences, Department of Animal Biosciences, University of Guelph, Guelph, ON N1G2W1, Canada; 3 Department of Animal Sciences, Laval University, Quebec, GC G1V0A6, Canada; 4 Faculdade de Ciências Agrárias e Veterinárias, Unesp Universidade Estadual Paulista, Jaboticabal 14884-900, SP, Brazil; luciano.hauschild@unesp.br; 5 School of Agronomy, Federal University of Rio Grande do Sul (UFRGS), Porto Alegre 91540-000, RS, Brazil; ines.andretta@ufrgs.br; 6 School of Veterinary Medicine and Animal Science, Federal University of Mato Grosso do Sul (UFMS), Campo Grande 79070-900, MS, Brazil; eduarda.b.xavier@ufms.br (E.B.X.); luis.itavo@ufms.br (L.C.V.I.)

**Keywords:** pig farming, precision feeding, precision nutrition, sustainability, livestock farming

## Abstract

This study comprehensively explores the environmental implications of two feeding strategies in pig farming, focusing on three scenarios: Brazilian tables (BT-2017), NRC (NRC-2012), and AGPIC (AGPIC-2021). The comparison involves conventional phase-feeding (CON) and the daily fit model (DFM). The five-phase system provided the same diet to all pigs within a group during each proposed phase. In contrast, the DFM adjusted the diet based on the nutritional requirements of pigs, anticipating subsequent diets through daily adjustments. We employed a cradle-to-gate approach, with the functional unit defined as one barrow with an initial body weight of 20.61 ± 0.85 kg, raised to 138.94 ± 0.90 kg over a 120-day growing-finishing period. Input data were sourced from observed commercial records from pig farms in Brazil, including over 1,000,000 data points from pigs raised under standard industry conditions. We evaluated the impact of the life cycle by considering factors such as acidification, climate change, ecotoxicity, eutrophication, land use, resource use, and water use. The OpenLCA software (version 1.11.0) and the Environmental Footprint 3.0 impact assessment method were used. Our results indicate that the DFM consistently outperforms the CON strategy in terms of reducing environmental impacts. Among the three scenarios, BT-2017 results in higher environmental impact reductions compared with NRC-2012 and AGPIC-2021. This is due to the higher concentration of corn and soybean meal in diets. Notable reductions include in relation to land use-related climate change impacts (12.55%), freshwater eutrophication (6.21%), mineral and metal resource depletion (6.11%), and fossil resource use (4.88%). These findings highlight that even modest adjustments to feeding strategies can effectively reduce the environmental footprint of pig farming.

## Simple Summary

Pig farming plays an important role in food production, but it also contributes to environmental impacts, such as greenhouse gas emissions, land and water use. We compared two pig feeding strategies to determine which is more environmentally friendly. The first, called the conventional phase-feeding system, provides all pigs with the same diet for several weeks. The second, called the daily fit model, adjusts the feed mixture daily to better meet the animals’ nutritional needs. Using real farm data from over one million pigs, the researchers evaluated how these systems impact climate change, water and land use, and resource depletion. The results showed that the daily feeding approach consistently lowered environmental impacts, particularly those related to land use, freshwater pollution, and fossil fuel consumption. Among the three feeding standards tested, the Brazilian feeding table achieved the greatest reductions. These findings demonstrate that small daily feed adjustments can substantially reduce the environmental footprint of pig production, helping farmers produce food more sustainably without compromising animal growth or health.

## 1. Introduction

Developing pig production worldwide has raised concerns regarding its environmental impact. Sustainable practices, particularly in waste management and feed production, are imperative to reduce the industry’s ecological footprint [1]. Despite the economic and social significance of swine farming, measures must be devised to maximize positive outcomes and minimize negative impacts. Thus, the development of the pig industry can assist in meeting sustainable development goals [2].

Despite its economic importance, pig farming is associated with substantial environmental impact, ranking it among the most polluting agricultural activities. This includes detrimental effects on air, water, and soil quality. Air quality is compromised through elevated emissions of ammonia, nitrous oxide, methane, and noxious odors [3,4]. High water consumption [5] and diminished water quality [3] further contribute to environmental strain. Excessive waste production [6] and inadequate treatment result in water eutrophication, altering aquatic biodiversity and fostering harmful organisms [7].

Feedstuff production primarily contributes to environmental impacts, such as climate change, acidification, and eutrophication. Thus, reducing the environmental impact of pig farming necessitates a focus on feed production. Growth performance, feeding formulas, and feeding plans significantly determine the environmental aspects of pig production systems [8,9]. Feeding strategies like the use of household waste and agro-industrial byproducts as feed ingredients [10], reduction in crude protein (CP) [11,12] and, supplementation with crystalline amino acids [11,13], alternative multi-objective formulation techniques [14], and precision feeding [15,16,17,18] can reduce environmental impacts and are suitable approaches to address the current challenges of animal production [14].

Precision feeding strategies can also reduce environmental impact and improve performance [19]. The modification of feeding management plays a central role. The conventional phase-feeding strategy uses predefined diets for each growth phase. In contrast, individual or group precision feeding uses adjusted diets dynamically tailored to more precisely meet the animal’s requirements. At the same time, different nutritional requirement guidelines like the Brazilian tables for pigs and poultry [20], the NRC for swine [21], and AGPIC [22] vary in their assumptions and formulations. These systems influence nutrient specifications and diet composition, affecting the environmental impact of pig production. Comparing these systems within different feeding strategies can provide insights into optimizing productivity and sustainability.

Based on these considerations, we hypothesize that the daily fit model (DFM) feeding strategy will reduce the environmental impact of growing-finishing pig production when compared to conventional phase-feeding strategies (CON). Therefore, this study aims to compare two feeding strategies for growing-finishing pigs, the CON and DFM, across three feeding formulation scenarios: BT-2017 [20], NRC-2012 [21], and AGPIC-2021 [22]. A life cycle analysis (LCA) was conducted to assess whether DFM can effectively reduce the environmental impact of a pig farm system across all feed formulation scenarios.

## 2. Materials and Methods

### 2.1. Simulation Study

In this simulation, data were collected from 11 feeding curves of pigs across four distinct sex categories (barrows, boars, females, and mixed groups), each with varying initial weights, average daily feed intakes (ADFI), and average daily gains (ADG). Specifically, growth data were obtained from barrows that started with an initial body weight of 20.61 ± 0.85 kg and reached a final body weight of 138.94 ± 0.90 kg over a 120-day growing-finishing period. The feed intake and growth curves were derived from a comprehensive database containing records of over 1,000,000 Landrace × Large White × Pietrain pigs, with commercial data provided by three operational pig farms. The pigs were maintained under commercial conditions, which included free access to water and feed, group housing, and an ambient room temperature of 20 to 24 °C, for 120 to 150 days.

This simulation employed three distinct scenarios based on the barrow requirements specified in the Brazilian tables for poultry and swine [20], the NRC for swine [21] and AGPIC [22]. The assessment compared two feeding models: the CON and the DFM. The CON provided the same diet to all pigs within a group during each proposed phase. In contrast, the DFM adjusted the diet based on pigs’ nutritional requirements, anticipating subsequent diets through daily adjustments.

The life cycle assessment was conducted using openLCA (version 1.11.0), an open-source software for LCA. This study used a net energy system that accounts for the energy available to the pig after metabolic losses and digestive processes. The simulation assumed a traditional Brazilian growing-finishing system, with group-housed pigs in controlled environments with ad libitum access to feed and water, and without applying a precision feeding strategy. In this simulation, two models are employed, each with distinct considerations. We systematically organized all the data collected to prepare them for detailed analysis and modeling using a deterministic approach. This study is based on established models and specific, predetermined inputs rather than the collection of new empirical data. In deterministic modeling, each set of input parameters produces a single, fixed output without probabilistic variation [23], meaning that outcomes are calculated directly from the model equations rather than through stochastic simulation or statistical inference. As this simulation employed predetermined parameters and mathematical equations to calculate environmental impacts, traditional statistical analyses such as analysis of variance or hypothesis testing were not applicable. More details of the model are explained in the first published article of this research [24].

Two models were used to compare the feed systems. The first model (1) calculates feed costs in the CON (CCF), considering the phase duration (D), feed price (F) during each phase, and feed intake in the respective phase (i). The total cost is determined by adding the costs of all phases, based on the number of feeding phases employed (Ph).

(1) $$CCF=\sum_{i=Ph}\left(D\times F\times I\right)$$

The second model (2) calculates daily feed costs by taking into account the cost of the feed used (CDA) and daily feed intake (DFI). Determining the total cost value (CTC) necessitates understanding the amount of feed intake (AFI) and the associated feed prices (FP) (3).

(2) $$CDA=\sum_{i=Ph}\left(CTC\times DFI\right)$$

(3) $$CTC=\left({AFI}_{1}\times {FP}_{1}\right)+\left({AFI}_{2}\times {FP}_{2}\right)$$

**Figure 1:**
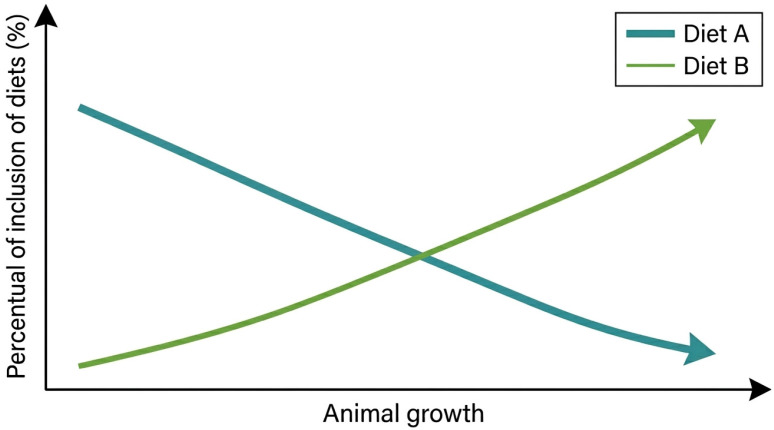
Visual description of the blending of the feed used in the daily fit model simulation.

The phase duration was expressed in days, and each phase had a defined daily feed intake (DFI) and associated feed price. For the DFM, two diets were blended during each phase, with the prices of feed one and feed two denoted as FP1 and FP2, respectively. The proportion of each feed was calculated for each day within the phase (d) using the equation PD = (100/d) × (D − 1), which determines the progressive transition from one feed to another. The amount of feed 1 (AFI1) was calculated as 100-percentage of diet (PD), and feed 2 (AFI2) as 100 − AFI1, ensuring a complete distribution of the 100% DFI. A visual description of this feed blending is presented in Figure 1. This modeling structure allowed for the dynamic adjustment of the diet composition across the feeding period, mimicking precision feeding strategies. It is a simple approach that can be developed and accessed using the Microsoft Excel spreadsheet.

### 2.2. Goal, Scope Definition, and System Boundary

This study used the CON and DFM to compare the environmental impact of three feeding scenarios for growing-finishing pigs: BT-2017, NRC-2012, and PIC-2021. The primary objective was to identify the feeding strategy with the lowest environmental impact.

**Figure 2:**
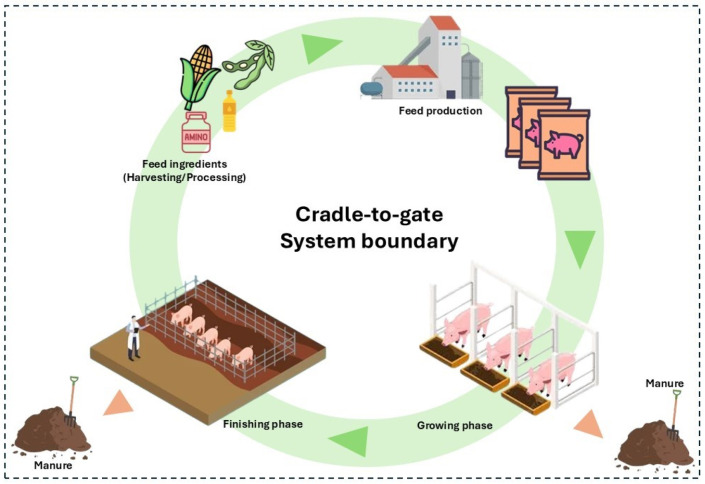
System boundary of pig production system.

This study adopts a cradle-to-gate approach, covering the stages of pig production until the farm gate. The functional unit is defined as one barrow with an initial weight of 20.61 ± 0.85 kg, reaching a final weight of 138.94 ± 0.90 kg over a 120-day growing-finishing period. This LCA excludes the impact of piglet production before 20.61 ± 0.85 kg, focusing solely on the growing-finishing phase. The system includes inputs such as piglets, feed, water, energy, transportation, and infrastructure components such as pig buildings, water consumption, and manure treatment via anaerobic digestion. The system’s boundaries encompass all processes on the pig farm, excluding veterinary products and care, artificial insemination, small cleaning materials, and processes beyond the farm gate, such as slaughtering and processing (Figure 2). Piglet production before 20.61 kg of live weight was excluded from the system boundary to focus on the growing-finishing phase, where the feeding strategies actually differ. The only difference between the two systems was the feeding strategy applied, and no other differences were expected, as previous studies have shown no significant effects on carcass yield or animal health [18,25,26].

### 2.3. Dataset Information and Scenario Development

The growth data from the pigs were obtained from a comprehensive database containing records of over 1,000,000 animals from three operational pig farms that provided commercial data. The LCA dataset utilized in this study, compiled in May 2020 using AGRIBALYSE V3.1, encompasses the entire life cycle of pig production, from the growing-finishing phase to departure from the farm to the slaughterhouse. The inventory spans 2018–2022, focusing on the technological aspects of the conventional output in standard pig breeding/fattening farms. The dataset includes comprehensive details of all activities on a pig farm, including inputs, infrastructure, emissions, and related buildings and barns.

Three scenarios, BT-2017, NRC-2012, and PIC-2021, were evaluated in the LCA, each comprising CON and DFM simulations. Diets were formulated at a minimal cost using solver procedures in Excel, following the nutritional requirements of Brazilian tables for swine and poultry [20], NRC [21] and AGPIC [22]. The scenarios were equivalent in all aspects except feed composition and quantity. The diet compositions and formulations are detailed in our previous study [24].

### 2.4. Feed Formulation and Composition

**Figure 3:**
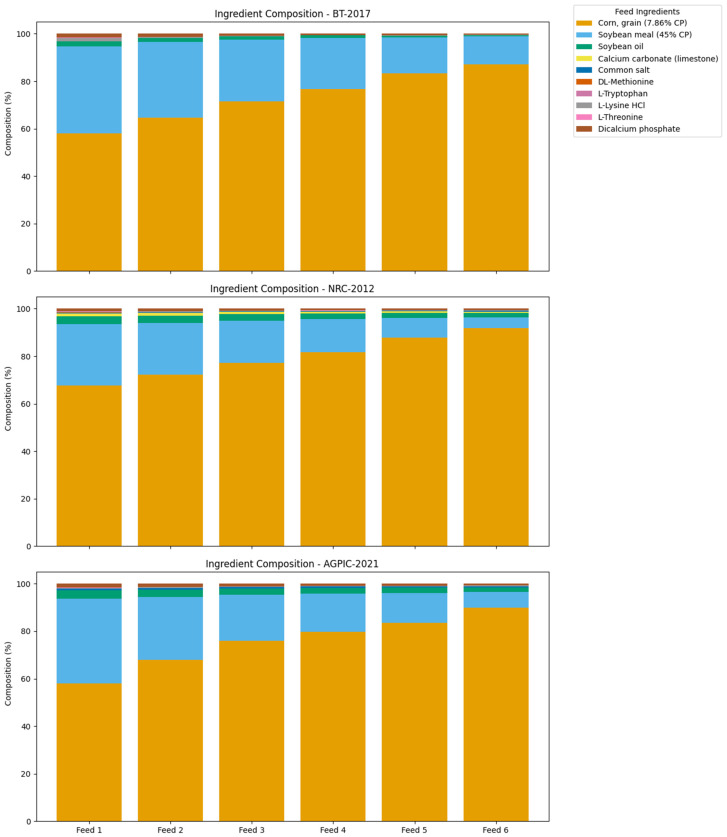
Ingredient composition (%) of feed formulations across six feeds for three nutritional requirement scenarios: Brazilian tables for pigs and poultry (BT-2017), National Research Council for swine (NRC-2012), and Agroceres PIC commercial guidelines (AGPIC-2021). Each bar represents the percentage inclusion of feed ingredients in the respective diet.

For each scenario, a total of six diets were formulated using the solver procedure in Microsoft Excel® (version 2307) and aligned with barrows’ nutrient requirements [24] (Supplementary Materials Tables S1 and S2). Five were used directly in the CON feeding system, aligning with the five predefined production phases. An additional sixth diet, called the ‘diluting diet’, was formulated with lower energy and CP levels and was used exclusively in the DFM strategy. This allowed for dynamic blending of two diets to adjust nutrient supply daily, according to predicted requirements. The quantity of each diet used in the simulation is provided in the Supplementary Materials, Table S3. Before the LCA, a modeling analysis assessed DFM’s cost reduction and nutrient excess compared to CON across all the scenarios. The inventory data reflect a Brazilian production scenario, particularly concerning the feed ingredients used, transportation distances, costs, and animal performance. The LCA aims to evaluate the environmental impact of these feeding strategies, considering the varying quantities used in each scenario based on a simulation study. The ingredient composition of each diet is shown in Figure 3.

### 2.5. Inventory Analysis and Impact Assessment Method

The inventory analysis used data from a standardized life cycle database, ensuring that the evaluated scenarios were representative. The life cycle inventory (LCI) phase involved compiling data on all relevant inputs and outputs, including raw material extraction, energy consumption, emissions, and waste generation. The data were sourced from recognized databases within OpenLCA, adhering to best practices for transparency and consistency. The selection of feed ingredients, transportation distances, and land use impacts was documented meticulously, as these factors significantly influence the results.

The transport distances were not adjusted for this study. All transport-related emissions were adopted directly from the AGRIBALYSE v1.4 database [24], which integrates average transport distances for each ingredient based on typical French and European supply chains. These embedded assumptions were maintained to ensure consistency and transparency in life cycle modeling. Importantly, the transport distances embedded in AGRIBALYSE are comparable to those reported by Villavicencio et al. [27,28] for Brazilian pig production systems, where average transport distances were around 350 km for corn, 450 km for soybean meal, and 250 km for amino acid supplements. This similarity supports the relevance of using AGRIBALYSE transport defaults even in a Brazilian production context, reducing the need for localization or adjustments. The allocation of environmental burdens in multi-functional processes, such as soybean processing, was carried out using a mass-based approach, consistent with the AGRIBALYSE methodology. Impacts were proportionally assigned to co-products (e.g., soybean meal and soybean oil) based on their mass outputs.

The impact assessment phase translated the LCI data into potential environmental impacts using the Environmental Footprint (EF) 3.0 method within OpenLCA. This method encompasses impact categories such as climate change (CC), eutrophication, acidification, and resource use, aligning with international LCA standards. The rationale for selecting this method was its robustness in relation to capturing key environmental aspects of feed production systems. Adjustments were made in impact categories to customize EF 3.0 for the context of swine production, considering factors such as feed type, waste management, and agricultural practices.

### 2.6. System Modeling and Compliance with Standards

The dataset creation employed the MEANS-InOut software version 5.4.3 (INRAE, Castanet-Tolosan, France) [29], incorporating methodologies like N_2_O—IPCC 2006 (Tier 2) [30,31], N—RMT 2016 [32], NO—EMEP/EEA 2016 (Tier 1) [33,34], CH_4_—IPCC 2006 [30,31], NO_3_—Basset-Mens 2007 [35], and NH_3_—EMEP 2009 (Tier 2) [33,34]. The modeling process ensured technological and geographical representativeness, maintained precision in parameter selection, and adhered to internationally recognized methodological standards. The LCA in this study analyzed parameters such as acidification, climate change, ecotoxicity, eutrophication, land use, resource use, and water use in each proposed scenario. Refer to Table 1 for detailed descriptions of all analyzed impact categories and their corresponding units.

The background data for the ingredients, transport, and energy inputs were obtained from the AGRIBALYSE LCI v1.4 database, which offers standardized, representative values for agricultural systems. This maintained consistency and methodological transparency across scenarios.

This LCA study adhered to ISO 14040/44 [36] standards, ensuring methodological rigor, comparability, and transparency in assessing the environmental impacts associated with swine feed production.

**Table 1:** Environmental impact categories analyzed in this study, their functional units, and their description.

Impact Category	Reference Unit	Description
Acidification	mol H+ eq	This indicates the potential acidification of soil and water due to the release of nitrogen and sulfur oxides.
Climate change	kg CO_2_ eq	Indicators of potential global warming due to emissions of greenhouse gases into the air, using carbon dioxide as a standard, with or without a change in land use.
Ecotoxicity of freshwater	CTUe	Impact of toxic substances emitted to the environment on freshwater organisms using the comparative toxic unit for ecosystems (CTUe) as a standard.
Eutrophication of freshwater	kg P eq	An indicator of the potential for increased phosphorus emissions to freshwater.
Eutrophication of marine water	kg N eq	An indicator of the potential for increased nitrogen emission to marine water.
Land use	Point	Impact of converting non-agricultural land into agricultural use.
Use of mineral and metal resources	kg Sb eq	An indicator of depletion of natural inorganic mineral and metal resources.
Use of fossil resources	MJ	Indicator of natural fossil fuel resource depletion in megajoules (MJ).
Water use	m3 depriv.	An indicator of the amount of water (cubic meters) used.

Note: All values derived using the Environmental Footprint (EF 3.0) method in OpenLCA. Adapted from Colomb et al. [37].

### 2.7. Uncertainty and Sensitivity Analysis

This study employed a deterministic modeling approach; the reliability of the percentage reduction estimates between DFM and CON strategies is subject to uncertainty arising from background inventory data. To address this limitation, a post hoc uncertainty and sensitivity analysis was conducted in Python (version 3.14.03) using only the life cycle impact assessment (LCIA) results generated from the OpenLCA.

Uncertainty was propagated through a Monte Carlo simulation (N = 10,000 iterations) applied to each LCIA result [23,38]. Each scenario value (DFM and CON) was modeled as a lognormal random variable, a distribution widely recommended for environmental inventory data due to its non-negativity and right-skewness [38,39,40]. The geometric standard deviation (GSD) used to parameterize the lognormal distributions was assigned per impact category following the Pedigree matrix approach of Weidema and Wesnaes [41], reflecting data quality indicators such as temporal, geographical, and technological representativeness. Values ranged from GSD = 1.05 for well-characterized categories (e.g., climate change from fossil resources) to GSD = 1.30 for categories with greater inherent variability (e.g., freshwater ecotoxicity). Because the DFM and CON systems share most background processes, differing only in feed quantity and blend proportion, a positive Pearson correlation coefficient of ρ = 0.85 was applied between paired DFM and CON samples within each scenario, following the approach of Heijungs and Huijbregts [42]. For each Monte Carlo iteration, the percentage reduction in environmental impact of DFM relative to CON was computed. From the resulting distribution, the 5th percentile (P5), median (P50), and 95th percentile (P95) were extracted to form a 90% confidence interval (CI) for each category-scenario combination. The probability that DFM resulted in a lower environmental impact than CON (i.e., a positive percentage reduction) was also computed.

A one-at-a-time (OAT) sensitivity analysis was performed to identify which input values most strongly influence the percentage reduction estimates [39,43]. Each LCIA result (DFM and CON separately) was perturbed by ±10%, ±20%, and ±30% while all other values were held constant, and the resulting change in the percentage reduction was recorded. A normalized sensitivity coefficient (Si) was calculated as

Si = Δ (% reduction)/Δ (% input)

where Δ (% reduction) is the absolute change in the percentage reduction, and Δ (% input) is the relative change in the perturbed input. The mean absolute Si across perturbation levels was used to rank categories by their sensitivity. All analyses were performed on the aggregated LCIA values reported in Supplementary Table S4.

## 3. Results

**Figure 4:**
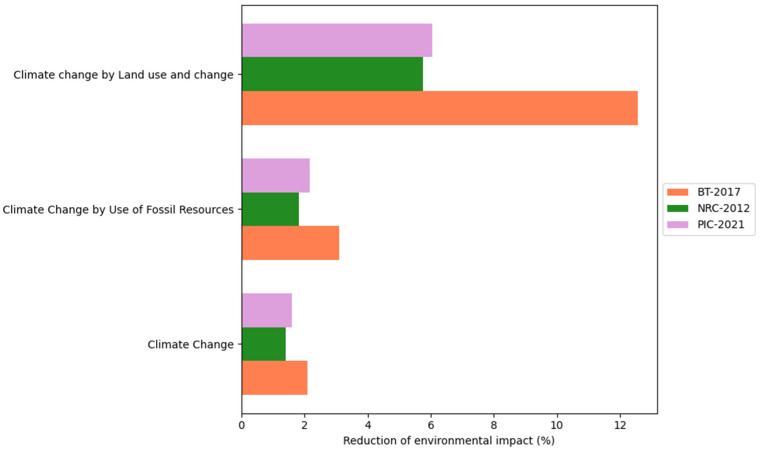
Percentage reduction in climate change (CC) and its subcategories, climate change from fossil resource use (CCFU) and climate change from land use and land use change (CCLU) when using the daily fit model (DFM) compared with the conventional phase-feeding system (CON) across the three nutritional scenarios.

**Figure 5:**
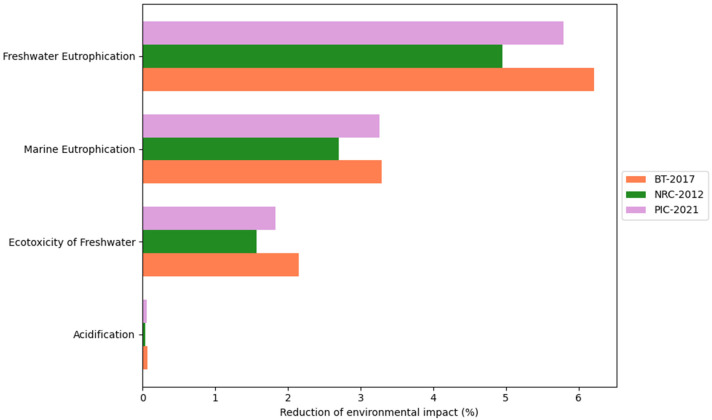
Percentage reduction in freshwater eutrophication, marine eutrophication, ecotoxicity of freshwater, and acidification when using the daily fit model (DFM) compared with the conventional phase-feeding system (CON) across the three nutritional scenarios.

**Figure 6:**
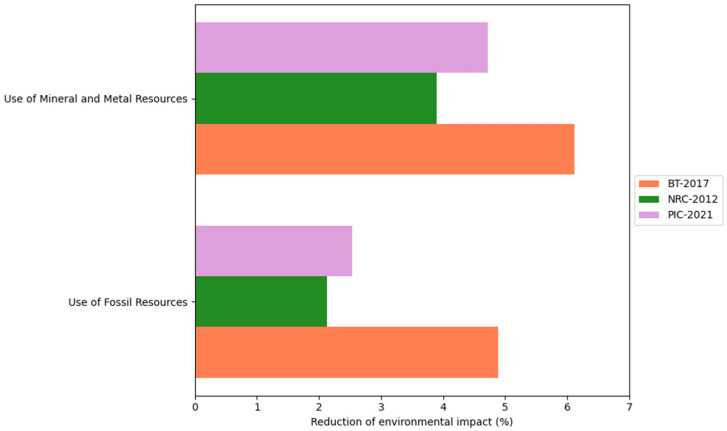
Percentage reduction in the use of mineral, metal, and fossil resources categories when using the daily fit model (DFM) compared with the conventional phase-feeding system (CON) across the three nutritional scenarios.

**Figure 7:**
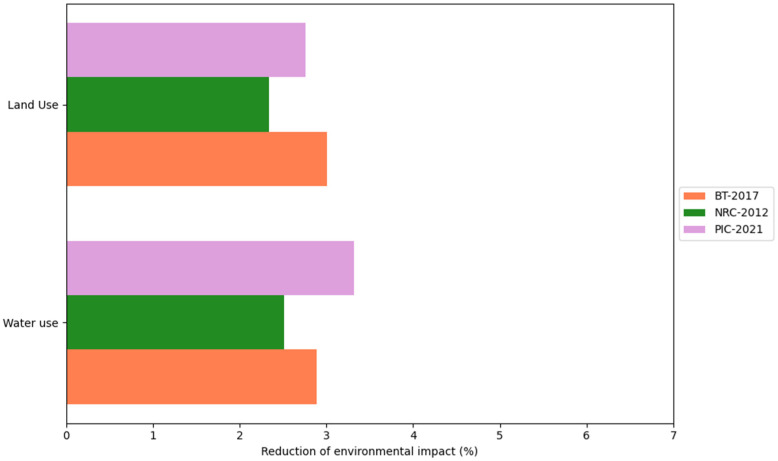
Percentage reduction in land use and water use impact categories when using the daily fit model (DFM) compared with the conventional phase-feeding system (CON) across the three nutritional scenarios.

This study considered the CON and DFM comparison for each of the three proposed scenarios: BT-2017, NRC-2012, and AGPIC-2021. Overall, the DFM consistently reduced environmental impact across all scenarios compared to the CON. For a detailed overview of the results, please refer to Supplementary Table S4. Additionally, Figure 4, Figure 5, Figure 6 and Figure 7 illustrate the percentage difference in reduced environmental impact when employing the DFM in a bar plot.

In the CC impact category, the evaluation focused on the global warming potential measured in radiative forcing over a 100-year horizon (kg CO_2_ equivalent). The CC caused by fossil resource use (CCFU) subcategory evaluates greenhouse gas emissions from burning fossil fuels. The CC caused by the land use and land use change (CCLU) subcategory evaluates the emissions and environmental impacts associated with changes in land use, mainly related to greenhouse gas emissions and their contribution to climate change [37].

Notably, the BT-2017 scenario exhibited higher reduction values for CC than the AGPIC-2021 and NRC-2012 scenarios, with respective values of 2.09% (from 42,177.46 to 41,291.90 kg CO_2_ eq), 1.59% (from 45,587.53 to 44,858.20 kg CO_2_ eq), and 1.41% (from 45,217.37 to 44,578.41 kg CO_2_ eq). The BT-2017 scenario also presented higher values for CCLU, with a 12.55% reduction (from 6127.30 to 5360.84 kg CO_2_ eq), followed by AGPIC-2021 and NRC-2012, with 6.03% (from 6309.91 to 5929.40 kg CO_2_ eq) and 5.75% (from 6247.52 to 5886.02 kg CO_2_ eq), respectively. For the CCFU subcategory, BT-2017 also exhibited higher reduction values of 3.10% (from 9523.82 to 9228.02 kg CO_2_ eq), followed by AGPIC-2021 (2.17%, from 10,140.58 to 9920.43 kg CO_2_ eq) and NRC-2012 (1.81%, from 9955.88 to 9775.80 kg CO_2_ eq). The reductions in the CC impact category and subcategories are presented in Figure 4.

Eutrophication categories assess phosphorus (P) and nitrogen (N) enrichment in marine and freshwater environments due to human activities [44]. Across all simulations, the values of eutrophication were lower in the DFM than in the CON. Specifically, reductions of 6.21% (from 1.77 to 1.66 kg P eq), 5.79% (from 1.87 to 1.76 kg P eq), and 4.95% (from 1.99 to 1.89 kg P eq) in freshwater eutrophication were observed in the BT-2017, AGPIC-2021, and NRC-2012 scenarios. Similarly, marine eutrophication reductions were noted at 3.28% (from 0.76 to 0.74 kg N eq), 3.26% (from 0.77 to 0.75 kg N eq), and 2.70% (from 0.80 to 0.78 kg N eq) for the exact scenarios. These findings suggest that the DFM effectively reduces nutrient levels such as P and N, thereby mitigating eutrophication impacts. Furthermore, the acidification impact category showed reductions of less than 1% for all the scenarios, indicating that the chosen system produces minimal amounts of acidifying substances (Figure 5).

The ecotoxicity of the freshwater impact category showed reductions of 2.15% (from 1,522,679.95 to 1,489,873.47 comparative toxic units for ecosystems (CTUe)), 1.83% (from 1,559,309.71 to 1,530,789.17 CTUe), and 1.57% (from 1,579,670.56 to 1,554,892.25 CTUe) in the respective BT-2017, AGPIC-2021, and NRC-2012 scenarios. These reductions signify a decrease in the potential adverse effects of products or processes on freshwater ecosystems and non-human organisms throughout their life cycle (Figure 5).

The use of mineral, metal and fossil resources throughout the LCA was measured. The resource use is measured in antimony equivalents, the use of minerals and metals, and the use of fossils is measured in megajoules to quantify fossil resource consumption. Regarding the use of mineral and metal resources, 6.11% (from 0.42 to 0.39 kg Sb eq), 4.72% (from 0.44 to 0.42 kg Sb eq), and 3.89% (from 0.46 to 0.44 kg Sb eq) reductions were achieved for the BT-2017, AGPIC-2021, and NRC-2012 scenarios, respectively. Similarly, reductions of 4.88% (from 764.16 to 727.86 MJ), 2.53% (from 789.08 to 769.13 MJ), and 2.13% (from 779.25 to 762.64 MJ) for the use of fossil resources were observed for the exact scenarios (Figure 6).

Water use evaluates the depletion of freshwater. Reductions of 3.32% (from 6921.79 to 6692.66 m^3^), 2.89% (from 7123.41 to 6917.37 m^3^), and 2.51% (from 7207.35 to 7026.36 m^3^) were found in the AGPIC-2021, BT-2017, and NRC-2012 scenarios. Land use assesses the impact of agriculture, settlements, and resource extraction on land. Reductions of 3.00% (from 1.26 to 1.22 Pt), 2.75% (from 1.31 to 1.27 Pt), and 2.33% (from 1.36 to 1.33 Pt) were found in the BT-2017, AGPIC-2021, and NRC-2012 scenarios, respectively (Figure 7).

**Figure 8:**
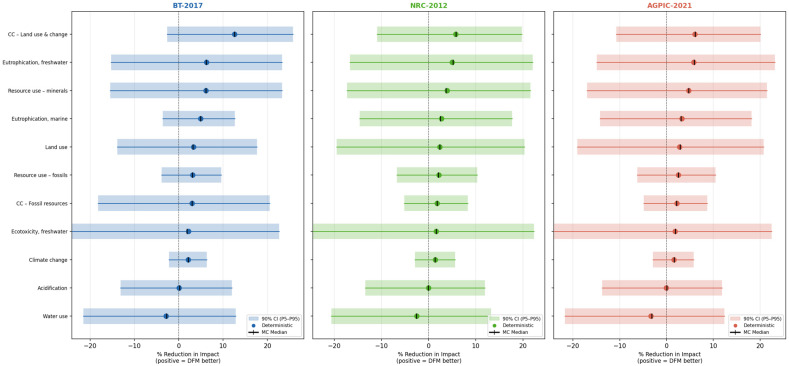
Monte Carlo forest plot showing 90% confidence intervals for percentage reduction in all impact categories and scenarios. Panels correspond to BT-2017, NRC-2012, and AGPIC-2021.

The Monte Carlo simulation confirmed that the direction of the DFM effect, with a lower environmental impact compared to CON, was robust across most impact categories and scenarios, although the width of the 90% CI varied considerably depending on the impact category (Figure 8; Table 2). For impact categories with a large deterministic reduction and relatively low background uncertainty, the CI remained clearly positive. In the BT-2017 scenario, CCLU showed the strongest statistical support, with a 91% probability that DFM outperformed CON (P5 = −2.6%, P50 = 12.6%, and P95 = 25.8%). Similarly, CCFU in BT-2017 returned a probability of 83% (P5 = −3.6%, P50 = 4.9%, and P95 = 12.6%), and CC reached 79% (P5 = −2.2%, P50 = 2.1%, and P95 = 6.3%).

In contrast, categories with small deterministic reductions and high background data uncertainty had wide CIs that included zero, indicating that the observed differences may not exceed background noise. Acidification showed near-zero deterministic reductions across all scenarios (<0.1%), and the Monte Carlo probability that DFM outperformed CON was approximately 51%, effectively indistinguishable from chance. Freshwater ecotoxicity returned probabilities ranging from 54% to 56% across scenarios, reflecting the high inherent uncertainty of ecotoxicity characterization factors (GSD = 1.30). Water use showed a negative deterministic reduction across all three scenarios, with Monte Carlo probabilities below 40%, confirming that this is a category where DFM did not provide a consistent environmental benefit.

Among the three nutritional scenarios, BT-2017 consistently returned the highest probability that DFM outperformed CON, averaging 67% across all categories. NRC-2012 returned the lowest average probability (60%), and AGPIC-2021 was intermediate (62%). This ordering mirrors the deterministic results and reflects the greater baseline environmental load of the BT-2017 formulation, which provides more scope for improvement under DFM.

The OAT sensitivity analysis revealed that the percentage reduction estimates were nearly equally sensitive to perturbations in the DFM and CON input values (Supplementary Figure S1; Table 2). Sensitivity coefficients (Si) were close to unity across most categories, indicating a near-linear relationship between input perturbations and changes in percentage reduction, consistent with the mathematical structure of a ratio-based metric. The highest mean absolute Si values were observed for water use (Si ≈ 1.08) and acidification (Si ≈ 1.05), both categories where the absolute difference between DFM and CON is small, making the ratio highly sensitive to small absolute changes in either value. Categories with large absolute differences, such as CCLU and freshwater eutrophication, showed lower relative sensitivity (Si ≈ 0.87–0.93), indicating more stable percentage reduction estimates. For more details on the results of the sensitivity analysis please refer to Supplementary Tables S5 and S6.

## 4. Discussion

### 4.1. Comparison of Scenarios

Evaluating the environmental impacts across scenarios, the DFM emerged as a compelling strategy for reducing environmental impacts compared to the CON approach. By isolating the feed system as the sole variable, the study provided a nuanced understanding of how feed management influences environmental outcomes throughout the pig production cycle. Notably, the growing-finishing phase, constituting over 72% of the time of the entire production cycle [45], is an important period where strategic feed interventions could effectively mitigate environmental impacts. This phase is particularly important due to the pigs’ higher weight at this time compared to in other feeding phases. With higher weights, their nutritional requirements, and amount of feed and manure increase [46,47]. Different feed and manure treatment strategies could be a great alternative to mitigate these effects.

Three different scenarios for nutrient requirements are also included in the feeding strategy. The differences in the results stem from the varied datasets used to develop their formulations and the methodological approaches employed to calculate their requirements. Formulations based on the Brazilian tables resulted in a higher inclusion of corn and soybean meals, as they required greater net energy and amino acids, leading to an increase in energy-dense ingredients. These tables were developed using empirical data from tropical conditions and modern genotypes raised in Brazilian production systems [20]. The NRC requirements have lower net energy and amino acid requirements levels, resulting in lower inclusion of energy-dense ingredients. The requirements were based on the United States of America’s data on their pig production systems, and a more conservative approach was used [21]. The Agroceres PIC requirements are a commercial guideline that combines the Brazilian and international data developed specifically for tropical regions. It aims to balance biological performance and cost-effectiveness, resulting in intermediate levels of nutrients [22].

One reason for the greater reduction in environmental impact under the BT-2017 scenario is how DFM interacts with the ingredient profile of the formulation. The BT-2017 diets included more corn, which is associated with significant greenhouse gas emissions and land use impacts. By reducing DFI variability, the DFM strategy lowers total feed input and, as a result, the environmental burden of these high-impact ingredients. In contrast, the NRC-2012 diets had lower nutrient densities and relied less on corn, leaving less room for improvement under DFM. This suggests that a formulation’s initial environmental load affects the potential gains from precision feeding, and that DFM strategies work better when applied to nutrient-dense, corn-rich diets.

Feed production is the major contributor to environmental impact, accounting for 60% of all emissions from the pig production supply chain [48]. McAuliffe et al. [48] also highlight that the processing of crop-based products, mainly corn and soybean, exerts a significant toll on various environmental categories like global warming, climate change, terrestrial acidification, marine eutrophication, biodiversity damage, and acidification [49,50]. In this context, precision feeding emerges, aiming to mitigate these impacts by reducing the excess nutrients in the pigs’ diets by adjusting to match their nutritional requirements more closely also to their actual requirements [15,26]. Other strategies, such as reducing CP levels and supplementing synthetic amino acids, have positive effects [11,12]. Other potential benefits include reduced feed costs and N and P excretion [25]. They are critical minerals, with a significant portion excreted in pig feces and urine. This minimizes environmental impacts without affecting the growth rate or feed efficiency of the pigs [51,52].

### 4.2. Reduction in Environmental Impact in the Impact Categories

Our results showed consistent reductions in all environmental indicators when applying the DFM strategy across all formulation scenarios, with the greatest improvements observed in BT-2017 due to its high baseline impact.

The category and subcategories of CC all resulted in a lower impact when using the DFM strategy. The reductions found in this study are similar to those in other LCAs, where the pig production system and feed production are the major contributors to CC. Specifically, pig housing accounts for approximately 30% of CC, whereas the feeding stage constitutes 63% of the overall value, with the latter being the primary influencer of CO_2_ emissions [47]. Notably, manure management further amplifies the pig farming CC contribution due to the emissions of pollutants like nitrate and nitrous oxide, associated with feed production and slurry excretion [53].

Reducing ingredients like corn and soybeans impacted CC, resulting in a lower overall environmental impact in all simulated scenarios. Similarly to Monteiro et al. [12,54], we observed that precision feeding strategies have reduced CC impact compared to conventional phase-feeding. Moreover, in other cases [12,38], decreasing CP in the diet, achieved by adding synthetic amino acids, has proven effective in diminishing CC impact, mainly when soybean meal linked to deforestation is used [12]. For instance, a 10 g/kg reduction in CP can generate a 101 kg CO_2_ eq decrease in the CC impact per ton of feed [10]. Although this study did not directly manipulate CP levels or supplement synthetic amino acids, the improved nutrient delivery in DFM indirectly reduces N and P excess, mimicking the environmental benefits observed in studies that apply CP reduction strategies [11,54].

Climate change by land use is strongly influenced by the cultivation of crops (e.g., corn and soybean), which are common in pigs’ diets. As noted by Alba-Reyes et al. [55] and Llorens et al. [56], the land transformation needed for corn and soybean cultivation accounts for 71% of CO_2_ emissions. Additionally, the transportation of feed ingredients, agricultural inputs, and animals contributes to the impact of CC by consuming fossil resources and increasing CO_2_ and CH_4_ emissions. Because the BT-2017 diets included more corn, a high-impact ingredient in terms of land use and global warming potential, their values were higher than those of the other two scenarios. Applying the DFM strategy in this scenario reduced overall feed use, significantly mitigating these land use-driven emissions. This emphasizes how dietary composition and ingredient sourcing critically affects the effectiveness of precision feeding strategies in reducing CC-related impacts.

In our results, the DFM led to eutrophication reductions of up to 6.21%, consistent with previous reports showing that reducing nutrient excess, particularly N and P, through more precise feeding or CP reduction strategies can yield eutrophication and acidification impact reductions of 5 to 11% [13,57].

The feed-related processes dominate freshwater eutrophication in pig production, ranging from 32% to 90% of the environmental impact [55,57,58], while approximately 88% of acidifying substances originate from the pig housing, particularly ammonia emissions [50]. The impact of eutrophication and acidification can be modulated through feed production, ingredient use, manure treatment, and feeding systems. Even small reductions in the inclusion of these main ingredients show benefits in obtaining lower eutrophication impact (mean of 16.3 g PO_4_–eq per kg of body weight gain) when compared with the conventional two-phase-feeding system (mean of 18.2 g PO_4_–eq per kg of body weight gain) [12].

With the reduction in nutrients in the DFM, the impact of ecotoxicity is also reduced. Pig manure significantly impacts ecotoxicity. The inclusion of chemicals like copper and zinc in pig diets, fertilizers, and pig manure significantly impact ecotoxicity [55,59,60]. The oversupply of these elements, leading to high concentrations in pig manure, can adversely affect freshwater organism populations through dose–response factors. An alternative approach involves substituting mineral fertilizers with digestate derived from pig manure, resulting in favorable environmental outcomes regarding terrestrial ecotoxicity, freshwater aquatic ecotoxicity, and marine aquatic ecotoxicity [61].

The same can be pointed out about using mineral, metal, and fossil resources, which have notable negative environmental impacts. Fertilizer production [62] and chemical methods to extract some compounds, such as phosphoric acid, that are commonly used in swine diets [63], and zinc [64], are integral to this impact category. Strategies to alleviate this impact include utilizing local feed ingredients, enhancing feed use, and improving manure management practices [65]. Precision feeding adjustments have demonstrated reductions in these impacts compared to conventional feeding systems.

Applying the DFM reduced water use across all scenarios, with the most significant reductions observed in the AGPIC-2021 and BT-2017 scenarios. This decrease is mainly due to the reduced total feed delivered under the DFM strategy and the lower inclusion of ingredients with high water footprints, such as corn. In this LCA, water use primarily refers to invisible or virtual water, that is, water embedded in the feed production process rather than direct animal consumption. The water used for drinking and washing in pig farming is negligible compared to the volume required for crop cultivation, particularly in relation to the irrigation of feed crops like corn and soybeans [66]. Diets such as BT-2017, which relied more heavily on corn, had higher baseline water use values. The DFM’s ability to reduce overall feed consumption helps mitigate this impact. Moreover, adopting alternative feed ingredients, such as agricultural byproducts, can further decrease water use due to their lower embedded water demands [67]. Precision feeding strategies like DFM, combined with alternative ingredient selection and improved nutrient delivery, contribute to more sustainable water resource use in pig production.

Precision feeding strategies like DFM mitigated the impact of land use by simply reducing the demand for feed. This optimization reduced excess nutrient intake, lowering the overall demand for feed ingredients. Land use impacts in pig production are largely driven by crop cultivation, particularly the land required to grow feed ingredients such as corn and soybean. These crops occupy significant agricultural areas and contribute heavily to the total land use footprint. Additionally, reducing CP levels in the diet has also been associated with reductions in land use. Studies have shown that such strategies can lead to a 5% decrease in land use values [11,12,15,68]. Thus, more efficient feed management and improved diet formulation are key tools for reducing the spatial footprint of pig production systems.

This simulation relied on modeled data and standard parameters from AGRIBALYSE, which may not capture regional variability across the entire system. It is a challenge to obtain detailed information on the cultivation, sourcing, and management of each ingredient, as well as on-farm practices to analyze the environmental impact accurately. Furthermore, the methodologies for measurements are also subject to scrutiny. Although three formulation scenarios were examined, they may not represent the full spectrum of global feed formulations.

The DFM strategy is not tied to a specific diet formulation but serves as a flexible nutritional approach that can be applied across various feeding programs. Given the diversity of pig production systems, regional variability in ingredient availability, and fluctuations in feed prices, no single formulation can be universally considered optimal. However, implementing an efficient feeding strategy like DFM is essential to reduce environmental impacts by improving nutrient delivery, minimizing excesses, and adapting to local constraints. This flexibility highlights the importance of aligning precision feeding techniques with context-specific nutritional requirements to achieve sustainable outcomes.

### 4.3. Uncertainty and Sensitivity of Results

The Monte Carlo simulation revealed an important distinction between impact categories in which the DFM advantage is robust and those in which it remains uncertain, given variability in background data. The most reliable findings were CCLU under BT-2017 (91% probability of DFM outperforming CON) and CCFU under BT-2017 (83%), which correspond precisely to the categories where the deterministic reductions were largest and where the ingredient profile of BT-2017 diets amplifies the sensitivity to feed quantity reduction. These results align with the broader LCA literature, in which the proportion of corn and soybean in feed formulations is consistently identified as the primary driver of climate change and land use impacts [59,60,65,66]. Because the DFM strategy reduces total feed quantity without altering the ingredient ratios within each diet, its environmental benefit scales proportionally with the initial environmental load of those ingredients. This explains why BT-2017, the most corn-intensive formulation, yielded the most statistically robust reductions.

The uncertainty results also provide important nuance for categories that appear beneficial under deterministic analysis, but whose CI overlap zero. Freshwater eutrophication, mineral resource use, and land use all showed positive deterministic reductions of 3–6%, but their 90% CIs spanned from approximately −15% to +23%, indicating that these estimates should be interpreted with caution. This wide interval arises from the relatively high uncertainty associated with P and metal characterization factors in the EF 3.0 method, a known limitation already documented [38,40,41]. This does not invalidate the directional finding that the DFM is expected to reduce these impacts, but it suggests that the magnitude of reduction may vary substantially depending on regional conditions, ingredient sourcing, and the specific characterization model applied.

The near-zero or negative deterministic reductions in acidification and water use, confirmed by Monte Carlo probabilities of approximately 50% and below 40%, respectively, warrant attention. Acidification is dominated by ammonia emissions from pig housing [60], a process that is identical across DFM and CON scenarios in this study’s system boundary, which explains why feed quantity adjustments have a negligible influence. The direction of the water use result, which shows CON with lower water use than DFM in all scenarios, reflects the virtual water content of feed ingredients rather than on-farm drinking water, and may be an artifact of the AGRIBALYSE background data representing French and European supply chains, where water-intensive irrigation patterns differ from Brazilian conditions [66]. These findings reinforce the importance of conducting regional water footprint assessments rather than relying on generic LCI databases to draw conclusions about water use.

The OAT sensitivity analysis showed that all percentage reduction estimates were nearly proportionally sensitive to changes in either the DFM or CON input values (Si ≈ 1.0 for most categories). This proportionality is expected mathematically when the metric of interest is the ratio of two values that differ by a small percentage. The practical implication is that the accuracy of LCIA resulting from the background database has a direct and nearly equal influence on comparisons between feeding strategies. Categories where the absolute difference between DFM and CON is small relative to the total impact, such as acidification and water use, show slightly higher sensitivity coefficients (Si > 1.0), meaning that even modest errors in background data could reverse the sign of the reduction estimate. This finding highlights that future studies comparing precision feeding strategies should prioritize improving the quality of life cycle inventory data for these high-sensitivity categories, particularly through region-specific characterization factors for water use and localized emission factors for acidifying compounds.

Taken together, the uncertainty and sensitivity analyses support the main conclusions of this study while appropriately qualifying their scope. The DFM strategy provides statistically robust environmental benefits for CC, CCFU, and CCLU, especially when applied to nutrient-dense, corn-intensive formulations such as BT-2017. For other categories, the directional benefit is plausible, but the magnitude is uncertain, and improvements in inventory data quality and regional parameterization would be needed to draw stronger conclusions. This graduated interpretation is consistent with the recommendations of the ISO 14044 standard, which emphasizes that LCA results should always be presented alongside an assessment of their uncertainty and limitations [36].

### 4.4. Practical Implications for Producers and Policymakers

A key practical advantage of the DFM is that it represents a nutritional management adjustment rather than a structural modification requiring capital investment in manure treatment or new infrastructure [15,18,25]. The strategy improves the daily match between nutrient supply and estimated animal requirements through feed blending, making it accessible to commercial farms. Importantly, the previous modeling phase of this research [24] indicated that DFM simultaneously reduces feed costs and nutrient excess, meaning that environmental improvement may align with economic benefit (a critical factor for producer adoption). The reductions observed here are also relevant from a policy perspective because they occur in impact categories commonly used to assess livestock sustainability, including CCLU (12.55% reduction in BT-2017), freshwater eutrophication (6.21%), fossil resource use (4.88%), and land use (3.00%) [46,55,56,68]. These categories are directly connected to broader regulatory concerns regarding feed sourcing, land occupation, nutrient losses, and dependence on non-renewable resources, which are increasingly relevant in environmental certification and agri-food supply chain reporting [48,59,64].

The practical significance of these findings increases when reductions are considered cumulatively across multiple impact categories and on a commercial scale. DFM consistently reduced environmental impacts across all three nutritional scenarios, with the BT-2017 formulation showing the greatest reductions due to its higher environmental load from corn- and soybean-based ingredients [55,56]. At the sectoral level, if 10% of Brazil’s annual pig slaughter (~5.8 million of 57.6 million head) [69] in the growing-finishing phase, DFM was adopted under the BT-2017 formulations, yielding annual reductions of approximately 3985 metric tons of CO_2_ eq. The reductions observed here align with earlier studies showing that precision feeding and crude protein reduction strategies can lower climate change, eutrophication, land use, and resource use impacts while maintaining animal performance [11,12,15,25,54,56], indicating that DFM is not only different from CON, but also practically relevant as a feasible strategy to support more sustainable pig production under commercial conditions.

## 5. Conclusions

Our simulations indicate consistent environmental benefits associated with the DFM strategy. Precision and alternative feeding techniques emerge as practical approaches to mitigate environmental impacts. Differences were found among the proposed scenarios (BT-2017, NRC-2012, and AGPIC-2021), primarily due to the modeling approach used to determine the nutrient requirements. While there is no single optimal recommendation, there are options to help mitigate these impacts. Adjustments to feed formulations to reduce their environmental impact can be made, especially by using alternative ingredients to corn and soybean meal.

A portion of the environmental benefits can already be achieved through simpler, easier, and more immediately applicable measures, such as daily adjustments by feeding groups, as simulated herein. A Monte Carlo uncertainty analysis and one-at-a-time sensitivity analysis further confirmed that the environmental benefits of the DFM strategy are robust for climate change from land use and fossil resource consumption, particularly under the BT-2017 scenario, where the probability of DFM outperforming CON exceeded 83% across these categories; however, categories such as acidification and water use showed wide confidence intervals that included zero, indicating that these estimates remain uncertain and dependent on the quality of background inventory data. Further mitigation potential exists through more advanced strategies, such as individual precision feeding, which could optimize nutrient utilization and reduce greenhouse gas emissions.

Overall, the study provides valuable insights into the environmental implications of different feeding models and scenarios, offering guidance for developing more sustainable and eco-friendly practices in the pig production industry. The results reinforce the importance of considering alternative feeding strategies and optimizing feed composition to minimize the environmental footprint of pig production.

## Figures and Tables

**Table 2:** Summary of Monte Carlo uncertainty analysis and one-at-a-time (OAT) sensitivity analysis for all impact categories across three nutritional scenarios.

Impact Category	Scenario	Det. % Red.	P5 (%)	P50 (%)	P95 (%)	Prob. DFM < CON (%)	Mean |Si|
Climate change	BT-2017	2.10	−2.2	2.1	6.3	78.9	1.00
Climate change	NRC-2012	1.41	−2.9	1.4	5.6	69.9	0.99
Climate change	AGPIC-2021	1.60	−2.9	1.6	5.8	72.2	0.99
CC—Land use and change	BT-2017	12.55	−2.6	12.6	25.8	91.3	0.90
CC—Land use and change	NRC-2012	5.75	−10.9	5.8	19.8	72.8	0.97
CC—Land use and change	AGPIC-2021	6.04	−10.7	6.1	20.1	73.3	0.96
CC—Fossil resources	BT-2017	3.11	−3.9	3.1	9.6	77.4	0.99
CC—Fossil resources	NRC-2012	1.81	−5.1	1.8	8.3	66.6	1.00
CC—Fossil resources	AGPIC-2021	2.18	−4.9	2.1	8.7	69.3	1.00
Eutrophication, freshwater	BT-2017	6.22	−15.3	6.2	23.3	69.6	0.96
Eutrophication, freshwater	NRC-2012	4.95	−16.7	5.1	22.1	66.3	0.97
Eutrophication, freshwater	AGPIC-2021	5.80	−14.9	6.0	23.2	68.8	0.97
Eutrophication, marine	BT-2017	3.29	−13.9	3.2	17.7	62.6	0.99
Eutrophication, marine	NRC-2012	2.70	−14.6	2.5	17.7	60.1	1.00
Eutrophication, marine	AGPIC-2021	3.27	−14.2	3.1	18.2	62.7	0.99
Ecotoxicity, freshwater	BT-2017	2.15	−24.1	1.8	22.6	55.2	0.99
Ecotoxicity, freshwater	NRC-2012	1.57	−24.5	1.6	22.3	54.1	1.00
Ecotoxicity, freshwater	AGPIC-2021	1.83	−24.1	1.9	22.5	55.3	0.99
Acidification	BT-2017	0.07	−13.1	0.2	12.0	51.2	1.02
Acidification	NRC-2012	−0.03	−13.4	−0.1	11.9	49.5	1.03
Acidification	AGPIC-2021	−0.06	−13.7	0.0	11.8	50.2	1.03
Resource use—minerals	BT-2017	6.12	−15.5	6.1	23.3	69.1	0.96
Resource use—minerals	NRC-2012	3.89	−17.3	3.7	21.5	61.8	0.99
Resource use—minerals	AGPIC-2021	4.71	−17.0	4.7	21.5	64.8	0.98
Resource use—fossils	BT-2017	4.89	−3.6	4.9	12.6	83.5	0.96
Resource use—fossils	NRC-2012	2.14	−6.7	2.1	10.3	65.3	1.00
Resource use—fossils	AGPIC-2021	2.54	−6.3	2.5	10.5	68.7	1.00
Water use	BT-2017	−2.89	−21.6	−2.8	12.9	38.7	1.05
Water use	NRC-2012	−2.51	−20.6	−2.6	13.2	39.9	1.05
Water use	AGPIC-2021	−3.32	−21.7	−3.2	12.4	37.8	1.09
Land use	BT-2017	3.00	−18.2	3.0	20.5	59.4	0.99
Land use	NRC-2012	2.34	−19.5	2.3	20.3	57.5	1.00
Land use	AGPIC-2021	2.76	−19.1	2.9	20.8	59.1	0.99

Note: P5, P50, and P95 denote the 5th, 50th, and 95th percentiles of the simulated percentage reduction in environmental impact (DFM vs. CON) from 10,000 Monte Carlo iterations. Prob. indicates the probability (%) that DFM resulted in a lower impact than CON. Mean |Si| is the mean absolute sensitivity coefficient across ±10%, ±20%, and ±30% perturbations of both DFM and CON input values. GSD values used in Monte Carlo simulation: 1.05 (CC—fossil resources), 1.08 (overall CC), 1.15 (acidification), 1.20 (CC—land use, eutrophication marine, and water use), 1.25 (eutrophication freshwater, mineral resources, and land use), and 1.30 (ecotoxicity freshwater). Correlation between DFM and CON: ρ = 0.85. Det. = deterministic value from OpenLCA.

## Data Availability

Complementary data are available in the tables and further information should be addressed to the corresponding author.

## Supplementary Materials

The following supporting information can be downloaded at: https://www.mdpi.com/article/10.3390/ani16101562/s1. Supplementary Table S1: Nutrient requirements for barrows used in the simulations; Supplementary Table S2: Formulation of the diets used in the simulations; Supplementary Table S3: Quantity of each feed used in the simulations; Supplementary Table S4: Impact categories of the Life Cycle Assessment; Supplementary Table S5: Detailed results of the one-at-a-time (OAT) sensitivity analysis for all impact categories across three nutritional scenarios (BT-2017, NRC-2012, AGPIC-2021); Supplementary Table S6: Ranking of impact categories by mean absolute sensitivity coefficient across three nutritional scenarios; and Supplementary Figure S1: One-at-a-time sensitivity ranges for percentage reduction in environmental impact.

## List of Abbreviations

ADF	average daily feed intake
ADG	average daily gain
AFI	amount of feed intake
CC	climate change
CCF	total feed cost in the conventional phase-feeding system
CCFU	climate change from fossil resource use
CCLU	climate change from land use and land use change
CDA	total feed cost in the daily fit model system
CI	confidence interval
CTC	cost of the daily feed mixture
CON	conventional phase-feeding system
CP	crude protein
CTUe	comparative toxic unit for ecosystems (freshwater ecotoxicity unit)
D	phase duration in days
d	day within the phase
DFI	daily feed intake
DFM	daily fit model
EF 3.0	environmental footprint 3.0 impact assessment method
F	feed price per unit for each diet used in blending
GSD	geometric standard deviation
IPCC	Intergovernmental Panel on Climate Change
LCA	life cycle assessment
LCI	life cycle inventory
MEANS-InOut	modular extrapolation of agricultural and food systems-input-output software
MC	Monte Carlo
MJ	megajoule
N	nitrogen
OAT	one-at-a-time
P	phosphorus
P5	5th percentile
P50	50th percentile (median)
P95	95th percentile
Ph	number of feeding phases
ρ	Pearson correlation coefficient
PD	percentage of diet transition (percentage of feed 2 in the daily blend)
Sb eq	antimony equivalents (unit for mineral and metal resource use)
Si	sensitivity coefficient
