# Supplementary material for: Reducing the Environmental Impact of Growing-Finishing Pig Production Through Daily Feed Adjustment: A Comparative Life Cycle Assessment
Source: Animals (Basel). 2026 May 21;16(10):1562. doi: 10.3390/ani16101562 (PMC13203624; doi:10.3390/ani16101562)
Supplement: Supplementary file 1 [file animals-16-01562-s001.zip › animals-4228482-supplementary.pdf]

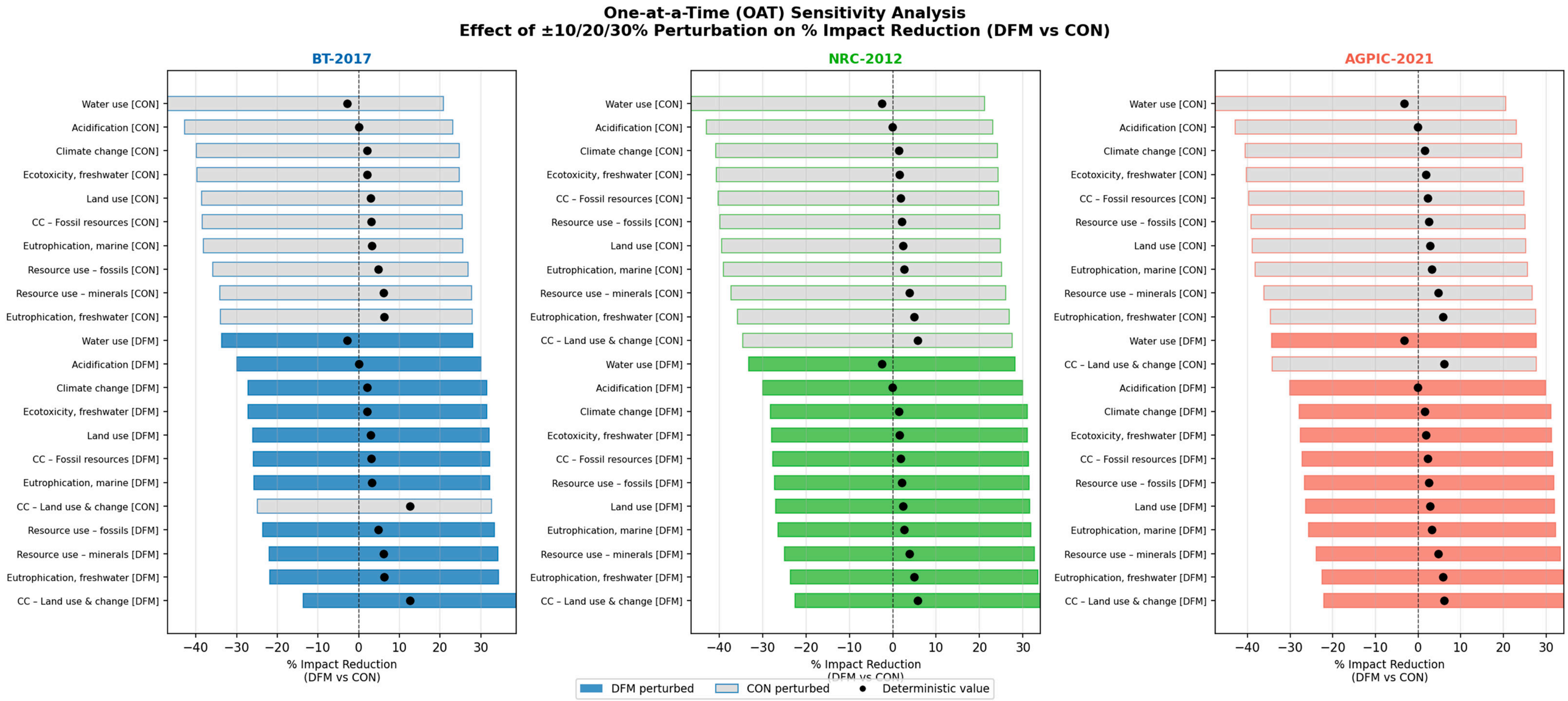

Supplementary Figure S1: One-at-a-time sensitivity ranges for percentage reduction in environmental impact when using the daily fit model (DFM) or conventional phase-feeding system (CON) across the three nutritional scenarios, Brazilian tables (BT-2017), NRC (NRC-2012), and AGPIC (AGPIC-2021).

Supplementary Table S1: Nutrient requirements for barrows used in the simulations.

Scenario	Brazilian Tables[1]						NRC[2]						AGPIC[3]					
Feed	1	2	3	4	5	6	1	2	3	4	5	6	1	2	3	4	5	6
Nutrients																		
ME	3350.000	3350.000	3350.000	3350.000	3350.000	3350.000	3400.000	3400.000	3400.000	3400.000	3400.000	3400.000	3400.000	3400.000	3400.000	3400.000	3400.000	3400.000
STTD P	0.421	0.346	0.279	0.241	0.215	0.183	0.330	0.300	0.260	0.240	0.210	0.200	0.420	0.360	0.300	0.270	0.250	0.220
SID Lysine	2.134	1.326	0.980	0.716	0.704	0.582	1.070	0.960	0.810	0.700	0.590	0.530	1.220	1.030	0.830	0.730	0.670	0.582
SID Methionine	0.363	0.321	0.278	0.242	0.209	0.196	0.310	0.280	0.230	0.200	0.170	0.150	-	-	-	-	-	-
SID Methionine + Cysteine	0.713	0.631	0.547	0.483	0.418	0.391	0.600	0.540	0.460	0.400	0.350	0.320	0.708	0.597	0.481	0.423	0.389	0.337
SID Threonine	0.813	0.695	0.603	0.523	0.453	0.419	0.600	0.580	0.500	0.450	0.390	0.360	0.793	0.670	0.540	0.482	0.442	0.384
SID Thyptophan	0.238	0.214	0.185	0.161	0.139	0.131	0.180	0.160	0.140	0.120	0.110	0.100	0.232	0.185	0.149	0.131	0.121	0.105
SID Valine	0.863	0.738	0.640	0.555	0.481	0.443	0.690	0.620	0.530	0.460	0.400	0.360	0.708	0.700	0.556	0.496	0.456	0.396
SID Isoleucine	0.688	0.588	0.510	0.443	0.383	0.353	0.560	0.500	0.430	0.370	0.320	0.290	0.683	0.577	0.465	0.409	0.375	0.326
SID Leucine	1.251	1.069	0.927	0.805	0.697	0.641	1.070	0.960	0.820	0.710	0.600	0.540	1.232	1.040	0.838	0.745	0.683	0.593
SID Histidine	0.413	0.353	0.306	0.266	0.230	0.210	0.370	0.330	0.280	0.240	0.200	0.180	0.403	0.350	0.282	0.248	0.228	0.198
SID Phenylalanine	0.626	0.535	0.464	0.403	0.349	0.323	0.640	0.570	0.490	0.420	0.360	0.330	-	-	-	-	-	-
SID Phenylalanine + Tyrosine	1.251	1.069	0.927	0.805	0.697	0.641	1.000	0.900	0.760	0.660	0.570	0.510	1.135	0.968	0.789	0.701	0.643	0.558

Abbreviations: ME = Metabolized Energy; SID = Standardized ileal digestibility.

Units: ME= kcal/kg; Other nutrients= %

[1]All requirements were obtained from (Rostagno, 2017)).

[2] All requirements were obtained from NRC (2012).

[3] All requirements were obtained from PIC (2021).

Supplementary Table S2: Formulation of the diets used in the simulations.

Diets																		
Scenario	Brazilian Tables						NRC						AGPIC					
Feed	1	2	3	4	5	6	1	2	3	4	5	6	1	2	3	4	5	6
Price of Feed*	$ 0.65	$ 0.64	$ 0.46	$ 0.45	$ 0.42	$ 0.40	$ 0.47	$ 0.46	$ 0.43	$ 0.41	$ 0.39	$ 0.38	$ 0.45	$ 0.42	$ 0.40	$ 0.38	$ 0.37	$ 0.35
Nutrients																		
ME	3297.305	3251.765	3241.603	3246.508	3209.390	3163.792	3400.000	3400.000	3400.000	3400.000	3400.000	3400.000	3400.000	3400.000	3400.000	3400.000	3400.000	3400.000
CP	19.120	19.539	15.960	13.964	11.676	8.938	17.883	16.486	14.998	13.596	12.539	11.650	18.375	16.919	16.393	13.932	11.308	10.168
Total Calcium	0.904	0.907	0.524	0.454	0.406	0.335	0.700	0.650	0.570	0.510	0.450	0.420	0.831	0.423	0.233	0.247	0.581	0.570
STTD P	0.403	0.405	0.254	0.254	0.197	0.162	0.330	0.300	0.260	0.240	0.210	0.200	0.420	0.360	0.300	0.270	0.250	0.220
SID Lysine	1.423	1.446	0.835	0.726	0.630	0.571	1.070	0.960	0.810	0.700	0.590	0.530	1.220	1.030	0.830	0.730	0.670	0.582
SID Methionine	0.442	0.448	0.251	0.218	0.189	0.198	0.343	0.296	0.230	0.212	0.200	0.190	0.440	0.346	0.232	0.205	0.203	0.168
SID Methionine + Cysteine	0.701	0.711	0.493	0.436	0.378	0.352	0.600	0.540	0.462	0.430	0.410	0.391	0.708	0.597	0.481	0.424	0.389	0.341
SID Threonine	0.891	0.905	0.543	0.472	0.410	0.263	0.600	0.580	0.500	0.450	0.390	0.360	1.339	0.787	0.540	0.482	0.817	0.770
SID Thyptophan	0.297	0.302	0.167	0.145	0.126	0.118	0.180	0.160	0.140	0.120	0.110	0.100	0.232	0.185	0.168	0.135	0.121	0.105
SID Valine	0.762	0.780	0.663	0.575	0.473	0.347	0.702	0.647	0.592	0.537	0.497	0.461	0.761	0.700	0.687	0.577	0.460	0.412
SID Isoleucine	0.695	0.713	0.584	0.495	0.391	0.264	0.648	0.587	0.527	0.466	0.422	0.383	0.683	0.621	0.607	0.494	0.375	0.326
SID Leucine	1.395	1.424	1.317	1.195	1.045	0.863	1.442	1.364	1.289	1.211	1.156	1.106	1.440	1.361	1.346	1.194	1.029	0.963
SID Histidine	0.438	0.448	0.393	0.346	0.290	0.222	0.434	0.403	0.372	0.341	0.319	0.299	0.443	0.412	0.406	0.347	0.282	0.257
SID Phenylalanine	0.807	0.826	0.702	0.607	0.496	0.361	0.773	0.710	0.647	0.584	0.538	0.497	0.804	0.742	0.729	0.609	0.479	0.426

SID Phenylalanine + Tyrosine	1.408	1.442	1.231	1.068	0.876	0.642	1.287	1.183	1.081	0.978	0.903	0.836	1.391	1.285	1.264	1.061	0.841	0.752
Total Nitrogen	2.655	2.716	2.232	1.939	1.605	1.204	2.861	2.638	2.400	2.175	2.006	1.864	2.739	2.463	2.293	1.934	1.570	1.384

Abbreviations: ME = Metabolized Energy; CP = Crude Protein; STTD P = Standardized Total Tract Digestible Phosphorus; SID = Standardized Ileal Digestibility.

Units: ME= kcal/kg; Other nutrients= %. * The conversion from Brazilian reais to US dollars was performed using an exchange rate of 5.05 reais per dollar.

Supplementary Table S3: Quantity of each feed used in the simulations

Quantity of feed (kg)	BT-2017 CON	BT-2017 DFM	NRC-2012 CON	NRC-2012 DFM	AGPIC-2021 CON	AGPIC-2021 DFM
Feed 1	27.879	13.07	27.879	13.07	27.879	13.07
Feed 2	53.919	41.12	53.919	41.12	53.919	41.12
Feed 3	69.381	62.50	69.381	62.50	69.381	62.50
Feed 4	42.318	56.81	42.318	56.81	42.318	56.81
Feed 5	60.167	51.36	60.167	51.36	60.167	51.36
Feed 6	-	28.80	-	28.80	-	28.80

*BT = Brazilian tables; CON = Conventional Model; DFM = Daily Fit Model;

Supplementary Table S4: Impact categories of the Life Cycle Assessment

Impact Category	Reference unit		BT-2017 DFM	BT-2017 CON	NRC-2012 DFM	NRC-2012 CON	AGPIC-2021 DFM	AGPIC-2021 CON
Acidification	mol H+ eq	Moles of Hydrogen Ion Equivalents	2,574227328	2,571463	2,574227	2,571463	2,56402	2,559329
Climate Change	kg CO2 eq	Kilograms of Carbon Dioxide Equivalents	161,9915526	166,7405	161,9916	166,7405	164,071	169,4916
Climate Change by Use of Fossil Resources	kg CO2 eq	Kilograms of Carbon Dioxide Equivalents	126,1468064	128,7102	126,1468	128,7102	128,6558	131,8016
Climate Change by Land Use	kg CO2 eq	Kilograms of Carbon Dioxide Equivalents	35,73510637	37,91548	35,73511	37,91548	35,30305	37,57201
Ecotoxicity of Freshwater	CTUe	Comparativ e Toxic Unit for Ecosystems	11439,08546	11627,69	11439,09	11627,69	11563,51	11785,96
Eutrophication of Freshwater	kg P eq	Kilograms of Phosphoru s Equivalents	0,072515531	0,076396	0,072516	0,076396	0,076953	0,081804
Eutrophication of Marine Water	kg N eq	Kilograms of Nitrogen Equivalents	1,484862737	1,530972	1,484863	1,530972	1,535101	1,59275
Land Use	Pt	Points	20143,5659	20629,2	20143,57	20629,2	20545,99	21132,77
Use of Fossil Resources	MJ	Megajoules	1468,452751	1506,757	1468,453	1506,757	1504,05	1550,71
Use of Minerals and Metals Resources	kg Sb eq	Kilograms of Antimony Equivalents	0,000327892	0,000342	0,000328	0,000342	0,000356	0,000375
Water Use	m3 depriv.	Cubic Meters of Water Deprivation	696,5331459	679,2064	696,5331	679,2064	675,0506	653,0025

- BT = Brazilian tables; CON = Conventional Model; DFM = Daily Fit Model.

Supplementary Table S5: Detailed results of the one-at-a-time (OAT) sensitivity analysis for all impact categories across three nutritional scenarios (BT-2017, NRC-2012, AGPIC-2021).

Impact category	Scenario	Parameter perturbed	Perturbation (%)	New % reduction	Δ % reduction	Sensitivity coefficient Si
Climate change	BT-2017	DFM	-30	31.4697	29.3701	-0.979
Climate change	BT-2017	DFM	-20	21.6797	19.5801	-0.979
Climate change	BT-2017	DFM	-10	11.8896	9.79	-0.979
Climate change	BT-2017	DFM	10	-7.6904	-9.79	-0.979
Climate change	BT-2017	DFM	20	-17.4805	-19.5801	-0.979
Climate change	BT-2017	DFM	30	-27.2705	-29.3701	-0.979
Climate change	BT-2017	CON	-30	-39.8577	-41.9573	1.3986
Climate change	BT-2017	CON	-20	-22.3755	-24.4751	1.2238
Climate change	BT-2017	CON	-10	-8.7782	-10.8778	1.0878
Climate change	BT-2017	CON	10	10.9996	8.9	0.89
Climate change	BT-2017	CON	20	18.4163	16.3167	0.8158
Climate change	BT-2017	CON	30	24.692	22.5924	0.7531
Climate change	NRC-2012	DFM	-30	30.9892	29.5761	-0.9859
Climate change	NRC-2012	DFM	-20	21.1305	19.7174	-0.9859
Climate change	NRC-2012	DFM	-10	11.2718	9.8587	-0.9859
Climate change	NRC-2012	DFM	10	-8.4456	-9.8587	-0.9859
Climate change	NRC-2012	DFM	20	-18.3043	-19.7174	-0.9859
Climate change	NRC-2012	DFM	30	-28.163	-29.5761	-0.9859
Climate change	NRC-2012	CON	-30	-40.8384	-42.2515	1.4084
Climate change	NRC-2012	CON	-20	-23.2336	-24.6467	1.2323
Climate change	NRC-2012	CON	-10	-9.541	-10.9541	1.0954
Climate change	NRC-2012	CON	10	10.3755	8.9624	0.8962
Climate change	NRC-2012	CON	20	17.8442	16.4312	0.8216
Climate change	NRC-2012	CON	30	24.1639	22.7508	0.7584
Climate change	AGPIC-2021	DFM	-30	31.1199	29.52	-0.984
Climate change	AGPIC-2021	DFM	-20	21.2799	19.68	-0.984
Climate change	AGPIC-2021	DFM	-10	11.4399	9.84	-0.984
Climate change	AGPIC-2021	DFM	10	-8.2402	-9.84	-0.984
Climate change	AGPIC-2021	DFM	20	-18.0802	-19.68	-0.984
Climate change	AGPIC-2021	DFM	30	-27.9202	-29.52	-0.984

Climate change	AGPIC-2021	CON	-30	-40.5716	-42.1715	1.4057
Climate change	AGPIC-2021	CON	-20	-23.0002	-24.6	1.23
Climate change	AGPIC-2021	CON	-10	-9.3335	-10.9334	1.0933
Climate change	AGPIC-2021	CON	10	10.5453	8.9455	0.8945
Climate change	AGPIC-2021	CON	20	17.9999	16.4	0.82
Climate change	AGPIC-2021	CON	30	24.3076	22.7077	0.7569
CC – Land use & change	BT-2017	DFM	-30	38.7862	26.2345	-0.8745
CC – Land use & change	BT-2017	DFM	-20	30.0414	17.4897	-0.8745
CC – Land use & change	BT-2017	DFM	-10	21.2965	8.7448	-0.8745
CC – Land use & change	BT-2017	DFM	10	3.8069	-8.7448	-0.8745
CC – Land use & change	BT-2017	DFM	20	-4.9379	-17.4897	-0.8745
CC – Land use & change	BT-2017	DFM	30	-13.6828	-26.2345	-0.8745
CC – Land use & change	BT-2017	CON	-30	-24.9261	-37.4778	1.2493
CC – Land use & change	BT-2017	CON	-20	-9.3104	-21.8621	1.0931
CC – Land use & change	BT-2017	CON	-10	2.8352	-9.7165	0.9716
CC – Land use & change	BT-2017	CON	10	20.5016	7.9498	0.795
CC – Land use & change	BT-2017	CON	20	27.1264	14.5747	0.7287
CC – Land use & change	BT-2017	CON	30	32.7321	20.1804	0.6727
CC – Land use & change	NRC-2012	DFM	-30	34.0253	28.2749	-0.9425
CC – Land use & change	NRC-2012	DFM	-20	24.6003	18.8499	-0.9425
CC – Land use & change	NRC-2012	DFM	-10	15.1754	9.425	-0.9425
CC – Land use & change	NRC-2012	DFM	10	-3.6746	-9.425	-0.9425
CC – Land use & change	NRC-2012	DFM	20	-13.0995	-18.8499	-0.9425
CC – Land use & change	NRC-2012	DFM	30	-22.5245	-28.2749	-0.9425
CC – Land use & change	NRC-2012	CON	-30	-34.6423	-40.3927	1.3464
CC – Land use & change	NRC-2012	CON	-20	-17.812	-23.5624	1.1781
CC – Land use & change	NRC-2012	CON	-10	-4.7218	-10.4722	1.0472
CC – Land use & change	NRC-2012	CON	10	14.3185	8.5681	0.8568
CC – Land use & change	NRC-2012	CON	20	21.4587	15.7083	0.7854
CC – Land use & change	NRC-2012	CON	30	27.5003	21.7499	0.725
CC – Land use & change	AGPIC-2021	DFM	-30	34.227	28.1884	-0.9396
CC – Land use & change	AGPIC-2021	DFM	-20	24.8308	18.7923	-0.9396
CC – Land use & change	AGPIC-2021	DFM	-10	15.4347	9.3961	-0.9396
CC – Land use & change	AGPIC-2021	DFM	10	-3.3576	-9.3961	-0.9396

CC – Land use & change	AGPIC-2021	DFM	20	-12.7537	-18.7923	-0.9396
CC – Land use & change	AGPIC-2021	DFM	30	-22.1499	-28.1884	-0.9396
CC – Land use & change	AGPIC-2021	CON	-30	-34.2306	-40.2692	1.3423
CC – Land use & change	AGPIC-2021	CON	-20	-17.4518	-23.4904	1.1745
CC – Land use & change	AGPIC-2021	CON	-10	-4.4016	-10.4402	1.044
CC – Land use & change	AGPIC-2021	CON	10	14.5805	8.5419	0.8542
CC – Land use & change	AGPIC-2021	CON	20	21.6988	15.6602	0.783
CC – Land use & change	AGPIC-2021	CON	30	27.722	21.6834	0.7228
CC – Fossil resources	BT-2017	DFM	-30	32.175	29.0678	-0.9689
CC – Fossil resources	BT-2017	DFM	-20	22.4858	19.3786	-0.9689
CC – Fossil resources	BT-2017	DFM	-10	12.7965	9.6893	-0.9689
CC – Fossil resources	BT-2017	DFM	10	-6.5821	-9.6893	-0.9689
CC – Fossil resources	BT-2017	DFM	20	-16.2714	-19.3786	-0.9689
CC – Fossil resources	BT-2017	DFM	30	-25.9606	-29.0678	-0.9689
CC – Fossil resources	BT-2017	CON	-30	-38.4183	-41.5255	1.3842
CC – Fossil resources	BT-2017	CON	-20	-21.116	-24.2232	1.2112
CC – Fossil resources	BT-2017	CON	-10	-7.6587	-10.7659	1.0766
CC – Fossil resources	BT-2017	CON	10	11.9156	8.8084	0.8808
CC – Fossil resources	BT-2017	CON	20	19.256	16.1488	0.8074
CC – Fossil resources	BT-2017	CON	30	25.4671	22.3599	0.7453
CC – Fossil resources	NRC-2012	DFM	-30	31.2691	29.4561	-0.9819
CC – Fossil resources	NRC-2012	DFM	-20	21.4505	19.6374	-0.9819
CC – Fossil resources	NRC-2012	DFM	-10	11.6318	9.8187	-0.9819
CC – Fossil resources	NRC-2012	DFM	10	-8.0056	-9.8187	-0.9819
CC – Fossil resources	NRC-2012	DFM	20	-17.8243	-19.6374	-0.9819
CC – Fossil resources	NRC-2012	DFM	30	-27.643	-29.4561	-0.9819
CC – Fossil resources	NRC-2012	CON	-30	-40.267	-42.0801	1.4027
CC – Fossil resources	NRC-2012	CON	-20	-22.7337	-24.5467	1.2273
CC – Fossil resources	NRC-2012	CON	-10	-9.0966	-10.9097	1.091
CC – Fossil resources	NRC-2012	CON	10	10.7392	8.9261	0.8926
CC – Fossil resources	NRC-2012	CON	20	18.1776	16.3645	0.8182
CC – Fossil resources	NRC-2012	CON	30	24.4716	22.6585	0.7553
CC – Fossil resources	AGPIC-2021	DFM	-30	31.5242	29.3468	-0.9782
CC – Fossil resources	AGPIC-2021	DFM	-20	21.7419	19.5645	-0.9782

CC – Fossil resources	AGPIC-2021	DFM	-10	11.9596	9.7823	-0.9782
CC – Fossil resources	AGPIC-2021	DFM	10	-7.6049	-9.7823	-0.9782
CC – Fossil resources	AGPIC-2021	DFM	20	-17.3872	-19.5645	-0.9782
CC – Fossil resources	AGPIC-2021	DFM	30	-27.1694	-29.3468	-0.9782
CC – Fossil resources	AGPIC-2021	CON	-30	-39.7466	-41.924	1.3975
CC – Fossil resources	AGPIC-2021	CON	-20	-22.2783	-24.4557	1.2228
CC – Fossil resources	AGPIC-2021	CON	-10	-8.6918	-10.8692	1.0869
CC – Fossil resources	AGPIC-2021	CON	10	11.0703	8.893	0.8893
CC – Fossil resources	AGPIC-2021	CON	20	18.4811	16.3038	0.8152
CC – Fossil resources	AGPIC-2021	CON	30	24.7518	22.5745	0.7525
Eutrophication, freshwater	BT-2017	DFM	-30	34.3525	28.1346	-0.9378
Eutrophication, freshwater	BT-2017	DFM	-20	24.9743	18.7564	-0.9378
Eutrophication, freshwater	BT-2017	DFM	-10	15.5961	9.3782	-0.9378
Eutrophication, freshwater	BT-2017	DFM	10	-3.1603	-9.3782	-0.9378
Eutrophication, freshwater	BT-2017	DFM	20	-12.5385	-18.7564	-0.9378
Eutrophication, freshwater	BT-2017	DFM	30	-21.9167	-28.1346	-0.9378
Eutrophication, freshwater	BT-2017	CON	-30	-33.9744	-40.1923	1.3397
Eutrophication, freshwater	BT-2017	CON	-20	-17.2276	-23.4455	1.1723
Eutrophication, freshwater	BT-2017	CON	-10	-4.2023	-10.4202	1.042
Eutrophication, freshwater	BT-2017	CON	10	14.7436	8.5256	0.8526
Eutrophication, freshwater	BT-2017	CON	20	21.8483	15.6303	0.7815
Eutrophication, freshwater	BT-2017	CON	30	27.8599	21.642	0.7214
Eutrophication, freshwater	NRC-2012	DFM	-30	33.4678	28.5138	-0.9505
Eutrophication, freshwater	NRC-2012	DFM	-20	23.9632	19.0092	-0.9505
Eutrophication, freshwater	NRC-2012	DFM	-10	14.4586	9.5046	-0.9505
Eutrophication, freshwater	NRC-2012	DFM	10	-4.5506	-9.5046	-0.9505
Eutrophication, freshwater	NRC-2012	DFM	20	-14.0552	-19.0092	-0.9505
Eutrophication, freshwater	NRC-2012	DFM	30	-23.5598	-28.5138	-0.9505
Eutrophication, freshwater	NRC-2012	CON	-30	-35.78	-40.734	1.3578
Eutrophication, freshwater	NRC-2012	CON	-20	-18.8075	-23.7615	1.1881
Eutrophication, freshwater	NRC-2012	CON	-10	-5.6067	-10.5607	1.0561
Eutrophication, freshwater	NRC-2012	CON	10	13.5945	8.6405	0.8641
Eutrophication, freshwater	NRC-2012	CON	20	20.795	15.841	0.7921
Eutrophication, freshwater	NRC-2012	CON	30	26.8877	21.9337	0.7311

Eutrophication, freshwater	AGPIC-2021	DFM	-30	34.0577	28.261	-0.942
Eutrophication, freshwater	AGPIC-2021	DFM	-20	24.6374	18.8407	-0.942
Eutrophication, freshwater	AGPIC-2021	DFM	-10	15.217	9.4203	-0.942
Eutrophication, freshwater	AGPIC-2021	DFM	10	-3.6236	-9.4203	-0.942
Eutrophication, freshwater	AGPIC-2021	DFM	20	-13.0439	-18.8407	-0.942
Eutrophication, freshwater	AGPIC-2021	DFM	30	-22.4643	-28.261	-0.942
Eutrophication, freshwater	AGPIC-2021	CON	-30	-34.5761	-40.3728	1.3458
Eutrophication, freshwater	AGPIC-2021	CON	-20	-17.7541	-23.5508	1.1775
Eutrophication, freshwater	AGPIC-2021	CON	-10	-4.6703	-10.467	1.0467
Eutrophication, freshwater	AGPIC-2021	CON	10	14.3607	8.5639	0.8564
Eutrophication, freshwater	AGPIC-2021	CON	20	21.4973	15.7005	0.785
Eutrophication, freshwater	AGPIC-2021	CON	30	27.5359	21.7392	0.7246
Eutrophication, marine	BT-2017	DFM	-30	32.3017	29.0136	-0.9671
Eutrophication, marine	BT-2017	DFM	-20	22.6305	19.3424	-0.9671
Eutrophication, marine	BT-2017	DFM	-10	12.9593	9.6712	-0.9671
Eutrophication, marine	BT-2017	DFM	10	-6.3831	-9.6712	-0.9671
Eutrophication, marine	BT-2017	DFM	20	-16.0543	-19.3424	-0.9671
Eutrophication, marine	BT-2017	DFM	30	-25.7255	-29.0136	-0.9671
Eutrophication, marine	BT-2017	CON	-30	-38.1599	-41.448	1.3816
Eutrophication, marine	BT-2017	CON	-20	-20.8899	-24.178	1.2089
Eutrophication, marine	BT-2017	CON	-10	-7.4577	-10.7458	1.0746
Eutrophication, marine	BT-2017	CON	10	12.0801	8.792	0.8792
Eutrophication, marine	BT-2017	CON	20	19.4068	16.1186	0.8059
Eutrophication, marine	BT-2017	CON	30	25.6062	22.3181	0.7439
Eutrophication, marine	NRC-2012	DFM	-30	31.8924	29.189	-0.973
Eutrophication, marine	NRC-2012	DFM	-20	22.1627	19.4593	-0.973
Eutrophication, marine	NRC-2012	DFM	-10	12.4331	9.7297	-0.973
Eutrophication, marine	NRC-2012	DFM	10	-7.0262	-9.7297	-0.973
Eutrophication, marine	NRC-2012	DFM	20	-16.7559	-19.4593	-0.973
Eutrophication, marine	NRC-2012	DFM	30	-26.4856	-29.189	-0.973
Eutrophication, marine	NRC-2012	CON	-30	-38.9951	-41.6985	1.39
Eutrophication, marine	NRC-2012	CON	-20	-21.6207	-24.3241	1.2162
Eutrophication, marine	NRC-2012	CON	-10	-8.1073	-10.8107	1.0811
Eutrophication, marine	NRC-2012	CON	10	11.5486	8.8451	0.8845

Eutrophication, marine	NRC-2012	CON	20	18.9195	16.2161	0.8108
Eutrophication, marine	NRC-2012	CON	30	25.1565	22.4531	0.7484
Eutrophication, marine	AGPIC-2021	DFM	-30	32.2856	29.0204	-0.9673
Eutrophication, marine	AGPIC-2021	DFM	-20	22.6121	19.347	-0.9673
Eutrophication, marine	AGPIC-2021	DFM	-10	12.9387	9.6735	-0.9673
Eutrophication, marine	AGPIC-2021	DFM	10	-6.4083	-9.6735	-0.9673
Eutrophication, marine	AGPIC-2021	DFM	20	-16.0818	-19.347	-0.9673
Eutrophication, marine	AGPIC-2021	DFM	30	-25.7553	-29.0204	-0.9673
Eutrophication, marine	AGPIC-2021	CON	-30	-38.1926	-41.4578	1.3819
Eutrophication, marine	AGPIC-2021	CON	-20	-20.9185	-24.1837	1.2092
Eutrophication, marine	AGPIC-2021	CON	-10	-7.4831	-10.7483	1.0748
Eutrophication, marine	AGPIC-2021	CON	10	12.0592	8.7941	0.8794
Eutrophication, marine	AGPIC-2021	CON	20	19.3876	16.1225	0.8061
Eutrophication, marine	AGPIC-2021	CON	30	25.5886	22.3234	0.7441
Ecotoxicity, freshwater	BT-2017	DFM	-30	31.5082	29.3536	-0.9785
Ecotoxicity, freshwater	BT-2017	DFM	-20	21.7236	19.5691	-0.9785
Ecotoxicity, freshwater	BT-2017	DFM	-10	11.9391	9.7845	-0.9785
Ecotoxicity, freshwater	BT-2017	DFM	10	-7.63	-9.7845	-0.9785
Ecotoxicity, freshwater	BT-2017	DFM	20	-17.4145	-19.5691	-0.9785
Ecotoxicity, freshwater	BT-2017	DFM	30	-27.1991	-29.3536	-0.9785
Ecotoxicity, freshwater	BT-2017	CON	-30	-39.7792	-41.9338	1.3978
Ecotoxicity, freshwater	BT-2017	CON	-20	-22.3068	-24.4614	1.2231
Ecotoxicity, freshwater	BT-2017	CON	-10	-8.7172	-10.8717	1.0872
Ecotoxicity, freshwater	BT-2017	CON	10	11.0496	8.895	0.8895
Ecotoxicity, freshwater	BT-2017	CON	20	18.4621	16.3076	0.8154
Ecotoxicity, freshwater	BT-2017	CON	30	24.7343	22.5797	0.7527
Ecotoxicity, freshwater	NRC-2012	DFM	-30	31.1012	29.5281	-0.9843
Ecotoxicity, freshwater	NRC-2012	DFM	-20	21.2585	19.6854	-0.9843
Ecotoxicity, freshwater	NRC-2012	DFM	-10	11.4158	9.8427	-0.9843
Ecotoxicity, freshwater	NRC-2012	DFM	10	-8.2695	-9.8427	-0.9843
Ecotoxicity, freshwater	NRC-2012	DFM	20	-18.1122	-19.6854	-0.9843
Ecotoxicity, freshwater	NRC-2012	DFM	30	-27.9549	-29.5281	-0.9843
Ecotoxicity, freshwater	NRC-2012	CON	-30	-40.6098	-42.1829	1.4061
Ecotoxicity, freshwater	NRC-2012	CON	-20	-23.0336	-24.6067	1.2303

Ecotoxicity, freshwater	NRC-2012	CON	-10	-9.3632	-10.9363	1.0936
Ecotoxicity, freshwater	NRC-2012	CON	10	10.5211	8.9479	0.8948
Ecotoxicity, freshwater	NRC-2012	CON	20	17.9776	16.4045	0.8202
Ecotoxicity, freshwater	NRC-2012	CON	30	24.287	22.7139	0.7571
Ecotoxicity, freshwater	AGPIC-2021	DFM	-30	31.2819	29.4506	-0.9817
Ecotoxicity, freshwater	AGPIC-2021	DFM	-20	21.465	19.6337	-0.9817
Ecotoxicity, freshwater	AGPIC-2021	DFM	-10	11.6482	9.8169	-0.9817
Ecotoxicity, freshwater	AGPIC-2021	DFM	10	-7.9856	-9.8169	-0.9817
Ecotoxicity, freshwater	AGPIC-2021	DFM	20	-17.8024	-19.6337	-0.9817
Ecotoxicity, freshwater	AGPIC-2021	DFM	30	-27.6193	-29.4506	-0.9817
Ecotoxicity, freshwater	AGPIC-2021	CON	-30	-40.241	-42.0723	1.4024
Ecotoxicity, freshwater	AGPIC-2021	CON	-20	-22.7109	-24.5422	1.2271
Ecotoxicity, freshwater	AGPIC-2021	CON	-10	-9.0763	-10.9076	1.0908
Ecotoxicity, freshwater	AGPIC-2021	CON	10	10.7557	8.9244	0.8924
Ecotoxicity, freshwater	AGPIC-2021	CON	20	18.1928	16.3614	0.8181
Ecotoxicity, freshwater	AGPIC-2021	CON	30	24.4856	22.6543	0.7551
Acidification	BT-2017	DFM	-30	30.0479	29.9795	-0.9993
Acidification	BT-2017	DFM	-20	20.0547	19.9863	-0.9993
Acidification	BT-2017	DFM	-10	10.0615	9.9932	-0.9993
Acidification	BT-2017	DFM	10	-9.9248	-9.9932	-0.9993
Acidification	BT-2017	DFM	20	-19.918	-19.9863	-0.9993
Acidification	BT-2017	DFM	30	-29.9111	-29.9795	-0.9993
Acidification	BT-2017	CON	-30	-42.7595	-42.8278	1.4276
Acidification	BT-2017	CON	-20	-24.9146	-24.9829	1.2491
Acidification	BT-2017	CON	-10	-11.0352	-11.1035	1.1104
Acidification	BT-2017	CON	10	9.1531	9.0847	0.9085
Acidification	BT-2017	CON	20	16.7236	16.6553	0.8328
Acidification	BT-2017	CON	30	23.1295	23.0611	0.7687
Acidification	NRC-2012	DFM	-30	29.9758	30.0104	-1.0003
Acidification	NRC-2012	DFM	-20	19.9723	20.0069	-1.0003
Acidification	NRC-2012	DFM	-10	9.9689	10.0035	-1.0003
Acidification	NRC-2012	DFM	10	-10.038	-10.0035	-1.0003
Acidification	NRC-2012	DFM	20	-20.0415	-20.0069	-1.0003
Acidification	NRC-2012	DFM	30	-30.045	-30.0104	-1.0003

Acidification	NRC-2012	CON	-30	-42.9065	-42.872	1.4291
Acidification	NRC-2012	CON	-20	-25.0432	-25.0086	1.2504
Acidification	NRC-2012	CON	-10	-11.1495	-11.115	1.1115
Acidification	NRC-2012	CON	10	9.0595	9.0941	0.9094
Acidification	NRC-2012	CON	20	16.6379	16.6724	0.8336
Acidification	NRC-2012	CON	30	23.0503	23.0849	0.7695
Acidification	AGPIC-2021	DFM	-30	29.9587	30.0177	-1.0006
Acidification	AGPIC-2021	DFM	-20	19.9528	20.0118	-1.0006
Acidification	AGPIC-2021	DFM	-10	9.9469	10.0059	-1.0006
Acidification	AGPIC-2021	DFM	10	-10.0649	-10.0059	-1.0006
Acidification	AGPIC-2021	DFM	20	-20.0708	-20.0118	-1.0006
Acidification	AGPIC-2021	DFM	30	-30.0767	-30.0177	-1.0006
Acidification	AGPIC-2021	CON	-30	-42.9414	-42.8824	1.4294
Acidification	AGPIC-2021	CON	-20	-25.0737	-25.0147	1.2507
Acidification	AGPIC-2021	CON	-10	-11.1766	-11.1177	1.1118
Acidification	AGPIC-2021	CON	10	9.0373	9.0963	0.9096
Acidification	AGPIC-2021	CON	20	16.6175	16.6765	0.8338
Acidification	AGPIC-2021	CON	30	23.0316	23.0905	0.7697
Resource use – minerals	BT-2017	DFM	-30	34.2813	28.1651	-0.9388
Resource use – minerals	BT-2017	DFM	-20	24.893	18.7768	-0.9388
Resource use – minerals	BT-2017	DFM	-10	15.5046	9.3884	-0.9388
Resource use – minerals	BT-2017	DFM	10	-3.2722	-9.3884	-0.9388
Resource use – minerals	BT-2017	DFM	20	-12.6606	-18.7768	-0.9388
Resource use – minerals	BT-2017	DFM	30	-22.0489	-28.1651	-0.9388
Resource use – minerals	BT-2017	CON	-30	-34.1197	-40.2359	1.3412
Resource use – minerals	BT-2017	CON	-20	-17.3547	-23.4709	1.1735
Resource use – minerals	BT-2017	CON	-10	-4.3153	-10.4315	1.0432
Resource use – minerals	BT-2017	CON	10	14.6511	8.5349	0.8535
Resource use – minerals	BT-2017	CON	20	21.7635	15.6473	0.7824
Resource use – minerals	BT-2017	CON	30	27.7817	21.6655	0.7222
Resource use – minerals	NRC-2012	DFM	-30	32.7207	28.834	-0.9611
Resource use – minerals	NRC-2012	DFM	-20	23.1094	19.2226	-0.9611
Resource use – minerals	NRC-2012	DFM	-10	13.4981	9.6113	-0.9611
Resource use – minerals	NRC-2012	DFM	10	-5.7245	-9.6113	-0.9611

Resource use – minerals	NRC-2012	DFM	20	-15.3359	-19.2226	-0.9611
Resource use – minerals	NRC-2012	DFM	30	-24.9472	-28.834	-0.9611
Resource use – minerals	NRC-2012	CON	-30	-37.3046	-41.1914	1.373
Resource use – minerals	NRC-2012	CON	-20	-20.1415	-24.0283	1.2014
Resource use – minerals	NRC-2012	CON	-10	-6.7925	-10.6792	1.0679
Resource use – minerals	NRC-2012	CON	10	12.6243	8.7376	0.8738
Resource use – minerals	NRC-2012	CON	20	19.9057	16.0189	0.8009
Resource use – minerals	NRC-2012	CON	30	26.0668	22.18	0.7393
Resource use – minerals	AGPIC-2021	DFM	-30	33.2961	28.5874	-0.9529
Resource use – minerals	AGPIC-2021	DFM	-20	23.7669	19.0583	-0.9529
Resource use – minerals	AGPIC-2021	DFM	-10	14.2378	9.5291	-0.9529
Resource use – minerals	AGPIC-2021	DFM	10	-4.8204	-9.5291	-0.9529
Resource use – minerals	AGPIC-2021	DFM	20	-14.3496	-19.0583	-0.9529
Resource use – minerals	AGPIC-2021	DFM	30	-23.8787	-28.5874	-0.9529
Resource use – minerals	AGPIC-2021	CON	-30	-36.1305	-40.8391	1.3613
Resource use – minerals	AGPIC-2021	CON	-20	-19.1141	-23.8228	1.1911
Resource use – minerals	AGPIC-2021	CON	-10	-5.8792	-10.5879	1.0588
Resource use – minerals	AGPIC-2021	CON	10	13.3715	8.6628	0.8663
Resource use – minerals	AGPIC-2021	CON	20	20.5906	15.8819	0.7941
Resource use – minerals	AGPIC-2021	CON	30	26.699	21.9903	0.733
Resource use – fossils	BT-2017	DFM	-30	33.4222	28.5334	-0.9511
Resource use – fossils	BT-2017	DFM	-20	23.911	19.0222	-0.9511
Resource use – fossils	BT-2017	DFM	-10	14.3999	9.5111	-0.9511
Resource use – fossils	BT-2017	DFM	10	-4.6223	-9.5111	-0.9511
Resource use – fossils	BT-2017	DFM	20	-14.1334	-19.0222	-0.9511
Resource use – fossils	BT-2017	DFM	30	-23.6446	-28.5334	-0.9511
Resource use – fossils	BT-2017	CON	-30	-35.8731	-40.7619	1.3587
Resource use – fossils	BT-2017	CON	-20	-18.889	-23.7778	1.1889
Resource use – fossils	BT-2017	CON	-10	-5.6791	-10.5679	1.0568
Resource use – fossils	BT-2017	CON	10	13.5353	8.6465	0.8646
Resource use – fossils	BT-2017	CON	20	20.7407	15.8519	0.7926
Resource use – fossils	BT-2017	CON	30	26.8375	21.9487	0.7316
Resource use – fossils	NRC-2012	DFM	-30	31.4945	29.3595	-0.9786
Resource use – fossils	NRC-2012	DFM	-20	21.7081	19.573	-0.9786

Resource use – fossils	NRC-2012	DFM	-10	11.9216	9.7865	-0.9786
Resource use – fossils	NRC-2012	DFM	10	-7.6514	-9.7865	-0.9786
Resource use – fossils	NRC-2012	DFM	20	-17.4379	-19.573	-0.9786
Resource use – fossils	NRC-2012	DFM	30	-27.2244	-29.3595	-0.9786
Resource use – fossils	NRC-2012	CON	-30	-39.8071	-41.9421	1.3981
Resource use – fossils	NRC-2012	CON	-20	-22.3312	-24.4662	1.2233
Resource use – fossils	NRC-2012	CON	-10	-8.7388	-10.8739	1.0874
Resource use – fossils	NRC-2012	CON	10	11.0319	8.8968	0.8897
Resource use – fossils	NRC-2012	CON	20	18.4459	16.3108	0.8155
Resource use – fossils	NRC-2012	CON	30	24.7193	22.5842	0.7528
Resource use – fossils	AGPIC-2021	DFM	-30	31.777	29.2384	-0.9746
Resource use – fossils	AGPIC-2021	DFM	-20	22.0308	19.4923	-0.9746
Resource use – fossils	AGPIC-2021	DFM	-10	12.2847	9.7461	-0.9746
Resource use – fossils	AGPIC-2021	DFM	10	-7.2076	-9.7461	-0.9746
Resource use – fossils	AGPIC-2021	DFM	20	-16.9538	-19.4923	-0.9746
Resource use – fossils	AGPIC-2021	DFM	30	-26.6999	-29.2384	-0.9746
Resource use – fossils	AGPIC-2021	CON	-30	-39.2307	-41.7692	1.3923
Resource use – fossils	AGPIC-2021	CON	-20	-21.8269	-24.3654	1.2183
Resource use – fossils	AGPIC-2021	CON	-10	-8.2906	-10.8291	1.0829
Resource use – fossils	AGPIC-2021	CON	10	11.3986	8.8601	0.886
Resource use – fossils	AGPIC-2021	CON	20	18.7821	16.2436	0.8122
Resource use – fossils	AGPIC-2021	CON	30	25.0296	22.4911	0.7497
Water use	BT-2017	DFM	-30	27.9766	30.8672	-1.0289
Water use	BT-2017	DFM	-20	17.6876	20.5781	-1.0289
Water use	BT-2017	DFM	-10	7.3985	10.2891	-1.0289
Water use	BT-2017	DFM	10	-13.1796	-10.2891	-1.0289
Water use	BT-2017	DFM	20	-23.4686	-20.5781	-1.0289
Water use	BT-2017	DFM	30	-33.7577	-30.8672	-1.0289
Water use	BT-2017	CON	-30	-46.9865	-44.0959	1.4699
Water use	BT-2017	CON	-20	-28.6132	-25.7226	1.2861
Water use	BT-2017	CON	-10	-14.3228	-11.4323	1.1432
Water use	BT-2017	CON	10	6.4631	9.3537	0.9354
Water use	BT-2017	CON	20	14.2579	17.1484	0.8574
Water use	BT-2017	CON	30	20.8534	23.744	0.7915

Water use	NRC-2012	DFM	-30	28.2421	30.7534	-1.0251
Water use	NRC-2012	DFM	-20	17.991	20.5023	-1.0251
Water use	NRC-2012	DFM	-10	7.7398	10.2511	-1.0251
Water use	NRC-2012	DFM	10	-12.7624	-10.2511	-1.0251
Water use	NRC-2012	DFM	20	-23.0135	-20.5023	-1.0251
Water use	NRC-2012	DFM	30	-33.2647	-30.7534	-1.0251
Water use	NRC-2012	CON	-30	-46.4447	-43.9334	1.4644
Water use	NRC-2012	CON	-20	-28.1391	-25.6278	1.2814
Water use	NRC-2012	CON	-10	-13.9014	-11.3901	1.139
Water use	NRC-2012	CON	10	6.8079	9.3192	0.9319
Water use	NRC-2012	CON	20	14.5739	17.0852	0.8543
Water use	NRC-2012	CON	30	21.1452	23.6565	0.7885
Water use	AGPIC-2021	DFM	-30	27.6747	30.9965	-1.0332
Water use	AGPIC-2021	DFM	-20	17.3425	20.6644	-1.0332
Water use	AGPIC-2021	DFM	-10	7.0104	10.3322	-1.0332
Water use	AGPIC-2021	DFM	10	-13.654	-10.3322	-1.0332
Water use	AGPIC-2021	DFM	20	-23.9862	-20.6644	-1.0332
Water use	AGPIC-2021	DFM	30	-34.3184	-30.9965	-1.0332
Water use	AGPIC-2021	CON	-30	-47.6026	-44.2808	1.476
Water use	AGPIC-2021	CON	-20	-29.1523	-25.8305	1.2915
Water use	AGPIC-2021	CON	-10	-14.802	-11.4802	1.148
Water use	AGPIC-2021	CON	10	6.0711	9.3929	0.9393
Water use	AGPIC-2021	CON	20	13.8985	17.2203	0.861
Water use	AGPIC-2021	CON	30	20.5217	23.8435	0.7948
Land use	BT-2017	DFM	-30	32.1014	29.0994	-0.97
Land use	BT-2017	DFM	-20	22.4016	19.3996	-0.97
Land use	BT-2017	DFM	-10	12.7018	9.6998	-0.97
Land use	BT-2017	DFM	10	-6.6978	-9.6998	-0.97
Land use	BT-2017	DFM	20	-16.3976	-19.3996	-0.97
Land use	BT-2017	DFM	30	-26.0974	-29.0994	-0.97
Land use	BT-2017	CON	-30	-38.5686	-41.5706	1.3857
Land use	BT-2017	CON	-20	-21.2475	-24.2495	1.2125
Land use	BT-2017	CON	-10	-7.7756	-10.7776	1.0778
Land use	BT-2017	CON	10	11.82	8.818	0.8818

Land use	BT-2017	CON	20	19.1683	16.1663	0.8083
Land use	BT-2017	CON	30	25.3862	22.3842	0.7461
Land use	NRC-2012	DFM	-30	31.6358	29.299	-0.9766
Land use	NRC-2012	DFM	-20	21.8695	19.5326	-0.9766
Land use	NRC-2012	DFM	-10	12.1031	9.7663	-0.9766
Land use	NRC-2012	DFM	10	-7.4295	-9.7663	-0.9766
Land use	NRC-2012	DFM	20	-17.1958	-19.5326	-0.9766
Land use	NRC-2012	DFM	30	-26.9621	-29.299	-0.9766
Land use	NRC-2012	CON	-30	-39.5188	-41.8557	1.3952
Land use	NRC-2012	CON	-20	-22.079	-24.4158	1.2208
Land use	NRC-2012	CON	-10	-8.5147	-10.8515	1.0851
Land use	NRC-2012	CON	10	11.2153	8.8785	0.8878
Land use	NRC-2012	CON	20	18.614	16.2772	0.8139
Land use	NRC-2012	CON	30	24.8745	22.5377	0.7513
Land use	AGPIC-2021	DFM	-30	31.9297	29.173	-0.9724
Land use	AGPIC-2021	DFM	-20	22.2054	19.4487	-0.9724
Land use	AGPIC-2021	DFM	-10	12.481	9.7243	-0.9724
Land use	AGPIC-2021	DFM	10	-6.9676	-9.7243	-0.9724
Land use	AGPIC-2021	DFM	20	-16.6919	-19.4487	-0.9724
Land use	AGPIC-2021	DFM	30	-26.4163	-29.173	-0.9724
Land use	AGPIC-2021	CON	-30	-38.919	-41.6757	1.3892
Land use	AGPIC-2021	CON	-20	-21.5541	-24.3108	1.2155
Land use	AGPIC-2021	CON	-10	-8.0481	-10.8048	1.0805
Land use	AGPIC-2021	CON	10	11.597	8.8403	0.884
Land use	AGPIC-2021	CON	20	18.9639	16.2072	0.8104
Land use	AGPIC-2021	CON	30	25.1975	22.4408	0.748

- Each row represents a single perturbation experiment. Impact category denotes the environmental impact category evaluated (e.g., Climate change, Eutrophication). Scenario refers to the nutritional formulation scenario (BT-2017; NRC-2012; AGPIC-2021). Parameter perturbed indicates whether the Daily Fit Model (DFM) or conventional phase-feeding (CON) LCIA result was modified. Perturbation (%) shows the percentage change applied to the input value (−30%, −20%, −10%, +10%, +20%, +30%). New % reduction is the recalculated percentage reduction in environmental impact (DFM vs. CON) after the perturbation. Δ % reduction represents the absolute change in percentage reduction compared to the baseline deterministic value. Sensitivity coefficient (Si) is the normalized sensitivity coefficient, calculated as the ratio of the relative change in percentage reduction to the relative change in the perturbed input. Values near ±1.0 indicate proportional sensitivity; values >1.0 indicate amplified sensitivity. CC = Climate change.

Supplementary Table S6: Ranking of impact categories by mean absolute sensitivity coefficient across three nutritional scenarios.

Category	Scenario	Mean |Si|
**Water use**	AGPIC-2021	1.0592
**Water use**	BT-2017	1.0547
**Water use**	NRC-2012	1.0509
**Acidification**	AGPIC-2021	1.0257
**Acidification**	NRC-2012	1.0255
**Acidification**	BT-2017	1.0244
**Climate change**	NRC-2012	1.0106
**Ecotoxicity, freshwater**	NRC-2012	1.009
**Climate change**	AGPIC-2021	1.0087
**CC – Fossil resources**	NRC-2012	1.0065
**Ecotoxicity, freshwater**	AGPIC-2021	1.0063
**Climate change**	BT-2017	1.0036
**Resource use – fossils**	NRC-2012	1.0032
**Ecotoxicity, freshwater**	BT-2017	1.003
**CC – Fossil resources**	AGPIC-2021	1.0028
**Land use**	NRC-2012	1.0012
**Resource use – fossils**	AGPIC-2021	0.9991
**Eutrophication, marine**	NRC-2012	0.9974
**Land use**	AGPIC-2021	0.9969
**Land use**	BT-2017	0.9943
**CC – Fossil resources**	BT-2017	0.9933
**Eutrophication, marine**	AGPIC-2021	0.9916
**Eutrophication, marine**	BT-2017	0.9914
**Resource use – minerals**	NRC-2012	0.9853
**Resource use – minerals**	AGPIC-2021	0.9768
**Resource use – fossils**	BT-2017	0.975
**Eutrophication, freshwater**	NRC-2012	0.9743
**CC – Land use & change**	NRC-2012	0.9662
**Eutrophication, freshwater**	AGPIC-2021	0.9657
**CC – Land use & change**	AGPIC-2021	0.9632
**Resource use – minerals**	BT-2017	0.9624

**Eutrophication, freshwater**	BT-2017	0.9614
**CC – Land use & change**	BT-2017	0.8964

- This table summarizes the sensitivity analysis by ranking all impact category-scenario combinations according to their mean absolute sensitivity coefficient (Mean |Si|). Category denotes the environmental impact category. Scenario indicates the nutritional formulation (BT-2017, NRC-2012, or AGPIC-2021). Mean |Si| is the mean absolute value of the sensitivity coefficient (Si) calculated across all perturbation levels (±10%, ±20%, ±30%) and both DFM and CON parameters. Higher values indicate greater sensitivity of the percentage-reduction estimate to changes in input data. Categories with Mean |Si| >1.0 are considered highly sensitive, meaning that small errors in LCIA input values could substantially affect the estimated environmental benefit of DFM. Categories are sorted in descending order of sensitivity. CC = Climate change.
